# Supplementary material for: Rapid detection and capture of clinical Escherichia coli strains mediated by OmpA-targeting nanobodies
Source: Commun Biol. 2025 Jul 14;8:1047. doi: 10.1038/s42003-025-08345-9 (PMC12259851; doi:10.1038/s42003-025-08345-9)
Supplement: Supplementary file 2 — Supplementary Information [file 42003_2025_8345_MOESM2_ESM.pdf]

Supplementary Information

Rapid detection and capture of clinical *Escherichia coli* strains mediated by OmpA-targeting nanobodies

Michèle Sorgenfrei[1], Lea M. Hürlimann[1], Andrea Printz[1,2], Fanny Wegner[1], Damien Morger[3], Fabian Ackle[1], Mélissa M. Remy[2], Grzegorz Montowski[3], Hans-Anton Keserue[3], Aline Cuénod[1], Frank Imkamp[1], Adrian Egli[1], Peter M. Keller[2,4], and Markus A. Seeger[1*]

[1]: Institute of Medical Microbiology, University of Zurich, Zurich, Switzerland

[2]: Institute for Infectious Diseases, University of Bern, Bern, Switzerland

[3]: rqmicro AG, Schlieren, Switzerland

[4]: current affiliation: Clinical Bacteriology / Mycology, University Hospital Basel, Basel, Switzerland

[*]Corresponding author: m.seeger@imm.uzh.ch

Table of contents

Supplementary Tables 1 – 11

Supplementary Figures 1 – 15

Supplementary Table 1 | Bacterial strains used in this study.

Clinical strain	OmpA isoform	Sample origin	Sero-group	OmpA protein sequence
E. coli MC1061 **(contains OmpA_short used for nanobody selections)**	short	reference	none (K-12)	**MKKTAIAIAVALAGFATVAQAAPKDNTWYTGAKLGWSQYHDTGFINNNGPTHE<br>NQLGAGAFGGYQVNPYVGFEMGYDWLGRMPYKGSVENGAYKAQGVQLTAKLGY<br>PITDDLDIYTRLGGMVWRADTKSNVYGKNHDTGVSPVFAGGVEYAITPEIATR<br>LEYQWTNNIGDAHTIGTRPDNGMLSLGVSYRFGQGEAAPVVAPAPAPAPEVQT<br>KHFTLKSDVLFNFNKATLKPEGQAALDQLYSQLSNLDPKDGSVVVLGYTDRIG<br>SDAYNQGLSERRAQSVVDYLISKGIPADKISARGMGESNPVTGNTCDNVKQRA<br>ALIDCLAPDRRVEIEVKGIKDVVTQPQA**
E. coli ATCC 11775 **(capsular K1 strain)**	short	reference	n/a	MKKTAIAIAVALAGFATVAQAAPKDNTWYTGAKLGWSQYHDTGFINNNGPTHE<br>NQLGAGAFGGYQVNPYVGFEMGYDWLGRMPYKGSVENGAYKAQGVQLTAKLGY<br>PITDDLDVYTRLGGMVWRADTKSNVYGKNHDTGVSPVFAGGVEYAITPEIATR<br>LEYQWTNNIGDAHTIGTRPDNGMLSLGVSYRFGQGEAAPVVAPAPAPAPEVQT<br>KHFTLKSDVLFTFNKATLKPEGQAALDQLYSQLSNLDPKDGSVVVLGYTDRIG<br>SDAYNQALSERRAQSVVDYLISKGIPADKISARGMGESNPVTGNTCDNVKQRA<br>ALIDCLAPDRRVEIEVKGIKDVVTQPQA
#1	short	Urine	O11	MKKTAIAIAVALAGFATVAQAAPKDNTWYTGAKLGWSQYHDTGFIPNNGPTHE<br>NQLGAGAFGGYQVNPYVGFEMGYDWLGRMPYKGDNINGAYKAQGVQLTAKLGY<br>PITDDLDIYTRLGGMVWRADTKANVPGGASFKDHDTGVSPVFAGGVEYAITPE<br>IATRLEYQWTNNIGDAHTIGTRPDNGMLSLGVSYRFGQGEVAPVVAPAPAPAP<br>EVQTKHFTLKSDVLFTFNKATLKPEGQAALDQLYSQLSNLDPKDGSVVVLGYT<br>DRIGSDAYNQALSERRAQSVVDYLISKGIPADKISARGMGESNPVTGNTCDNV<br>KQRAALIDCLAPDRRVEIEVKGIKDVVTQPQA
#2	short	Urine	O81	MKKTAIAIAVALAGFATVAQAAPKDNTWYTGAKLGWSQYHDTGFINNNGPTHE<br>NQLGAGAFGGYQVNPYVGFEMGYDWLGRMPYKGSVENGAYKAQGVQLTAKLGY<br>PITDDLDIYTRLGGMVWRADTKSNVYGKNHDTGVSPVFAGGVEYAITPEIATR<br>LEYQWTNNIGDAHTIGTRPDNGMLSLGVSYRFGQGEAAPVVAPAPAPAPEVQT<br>KHFTLKSDVLFNFNKATLKPEGQAALDQLYSQLSNLDPKDGSVVVLGYTDRIG<br>SDAYNQGLSERRAQSVVDYLISKGIPADKISARGMGESNPVTGNTCDNVKQRA<br>ALIDCLAPDRRVEIEVKGIKDVVTQPQA
#3	short	Vaginal swap	O4	MKKTAIAIAVALAGFATVAQAAPKDNTWYTGAKLGWSQYHDTGFINNNGPTHE<br>NQLGAGAFGGYQVNPYVGFEMGYDWLGRMPYKGSVENGAYKAQGVQLTAKLGY<br>PITDDLDVYTRLGGMVWRADTKSNVYGKNHDTGVSPVFAGGVEYAITPEIATR<br>LEYQWTNNIGDAHTIGTRPDNGMLSLGVSYRFGQGEAAPVVAPAPAPAPEVQT<br>KHFTLKSDVLFTFNKATLKPEGQAALDQLYSQLSNLDPKDGSVVVLGYTDRIG<br>SDAYNQALSERRAQSVVDYLISKGIPADKISARGMGESNPVTGNTCDNVKQRA<br>ALIDCLAPDRRVEIEVKGIKDVVTQPQA
#4	short	Urine	O134	MKKTAIAIAVALAGFATVAQAAPKDNTWYTGAKLGWSQYHDTGFIDNNGPTHE<br>NQLGAGAFGGYQVNPYVGFEMGYDWLGRMPYKGSVENGAYKAQGVQLTAKLGY<br>PITDDLDVYTRLGGMVWRADTKSNVYGKNHDTGVSPVFAGGVEYAITPEIATR<br>LEYQWTNNIGDAHTIGTRPDNGMLSLGVSYRFGQGEVAPVVAPAPAPAPEVQT<br>KHFTLKSDVLFTFNKATLKPEGQAALDQLYSQLSNLDPKDGSVVVLGYTDRIG<br>SDAYNQALSERRAQSVVDYLISKGIPADKISARGMGESNPVTGNTCDNVKQRA<br>ALIDCLAPDRRVEIEVKGIKDVVTQPQA
#5	long	Urine	O9	MKKTAIAIAVALAGFATVAQAAPKDNTWYTGAKLGWSQYHDTGFIPNNGPTHE<br>NQLGAGAFGGYQVNPYVGFEMGYDWLGRMPYKGDNINGAYKAQGVQLTAKLGY<br>PITDDLDVYTRLGGMVWRADTKANVPGGASFKDHDTGVSPVFAGGVEYAITPE<br>IATRLEYQWTNNIGDANTIGTRPDNGLLSLGVSYRFGQGEAAPVVAPAPAPAP<br>EVQTKHFTLKSDVLFNFNKATLKPEGQAALDQLYSQLSNLDPKDGSVVVLGYT<br>DRIGSDAYNQGLSERRAQSVVDYLISKGIPADKISARGMGESNPVTGNTCDNV<br>KQRAALIDCLAPDRRVEIEVKGIKDVVTQPQA
#6	long	n/a	O6	MKKTAIAIAVALAGFATVAQAAPKDNTWYTGAKLGWSQYHDTGFIPNNGPTHE<br>NQLGAGAFGGYQVNPYVGFEMGYDWLGRMPYKGDNINGAYKAQGVQLTAKLGY<br>PITDDLDIYTRLGGMVWRADTKANVPGGASFKDHDTGVSPVFAGGVEYAITPE<br>IATRLEYQWTNNIGDAHTIGTRPDNGMLSLGVSYRFGQGEVAPVVAPAPAPAP<br>EVQTKHFTLKSDVLFTFNKATLKPEGQAALDQLYSQLSNLDPKDGSVVVLGYT<br>DRIGSDAYNQALSERRAQSVVDYLISKGIPADKISARGMGESNPVTGNTCDNV<br>KQRAALIDCLAPDRRVEIEVKGIKDVVTQPQA
#7	long	Urine	O8	MKKTAIAIAVALAGFATVAQAAPKDNTWYTGAKLGWSQYHDTGFIPNNGPTHE<br>NQLGAGAFGGYQVNPYVGFEMGYDWLGRMPYKGDNINGAYKAQGVQLTAKLGY<br>PITDDLDVYTRLGGMVWRADTKANVPGGASFKDHDTGVSPVFAGGVEYAITPE<br>IATRLEYQWTNNIGDAHTIGTRPDNSMLSLGVSYRFGQGEAAPVVAPAPAPAP<br>EVQTKHFTLKSDVLFNFNKATLKPEGQAALDQLYSQLSNLDPKDGSVVVLGYT<br>DRIGSDAYNQGLSERRAQSVVDYLISKGIPADKISARGMGESNPVTGNTCDNV<br>KQRAALIDCLAPDRRVEIEVKGIKDVVTQPQA

Clinical strain	OmpA isoform	Sample origin	Sero-group	OmpA protein sequence
#8	long	Urine	O25	MKKTAIAIAVALAGFATVAQAAPKDNTWYTGAKLGWSQYHDTGFIPNNGPTHE<br>NQLGAGAFGGYQVNPYVGFEMGYDWLGRMPYKGDNINGAYKAQGVQLTAKLGY<br>PITDDLDIYTRLGGMVWRADTKANVPGGASFKDHDTGVSPVFAGGVEYAITPE<br>IATRLEYQWTNNIGDAHTIGTRPDNGMLSLGVSYRFGQGEAAPVVAPAPAPAP<br>EVQTKHFTLKSDVLFTFNKATLKPEGQAALDQLYSQLSNLDPKDGSVVVLGYT<br>DRIGSDAYNQALSERRAQSVVDYLISKGIPADKISARGMGESNPVTGNTCDNV<br>KQRAALIDCLAPDRRVEIEVKGIKDVVTQPQA
#9	long	Urine	O55	MKKTAIAIAVALAGFATVAQAAPKDNTWYTGAKLGWSQYHDTGFIPNNGPTHE<br>NQLGAGAFGGYQVNPYVGFEMGYDWLGRMPYKGDNINGAYKAQGVQLTAKLGY<br>PITDDLDVYTRLGGMVWRADTKANVPGGASFKDHDTGVSPVFAGGVEYAITPE<br>IATRLEYQWTNNIGDANTIGTRPDNGLLSLGVSYRFGQGEAAPVVAPAPAPAP<br>EVQTKHFTLKSDVLFNFNKATLKPEGQAALDQLYSQLSNLDPKDGSVVVLGYT<br>DRIGSDAYNQGLSERRAQSVVDYLISKGIPADKISARGMGESNPVTGNTCDNV<br>KQRAALIDCLAPDRRVEIEVKGIKDVVTQPQA
#10	long	Urine	O21	MKKTAIAIAVALAGFATVAQAAPKDNTWYTGAKLGWSQYHDTGFIPNNGPTHE<br>NQLGAGAFGGYQVNPYVGFEMGYDWLGRMPYKGDNINGAYKAQGVQLTAKLGY<br>PITDDLDVYTRLGGMVWRADTKANVPGGASFKDHDTGVSPVFAGGVEYAITPE<br>IATRLEYQWTNNIGDAHTIGTRPDNGMLSLGVSYRFGQGEAAPVVAPAPAPAP<br>EVQTKHFTLKSDVLFNFNKATLKPEGQAALDQLYSQLSNLDPKDGSVVVLGYT<br>DRIGSDAYNQGLSERRAQSVVDYLISKGIPADKISARGMGESNPVTGNTCDNV<br>KQRAALIDCLAPDRRVEIEVKGIKDVVTQPQA
#11 **(contains OmpA_long used for nanobody selections)**	long	Urine	O16	**MKKTAIAIAVALAGFATVAQAAPKDNTWYTGAKLGWSQYHDTGFIPNNGPTHE<br>NQLGAGAFGGYQVNPYVGFEMGYDWLGRMPYKGDNINGAYKAQGVQLTAKLGY<br>PITDDLDIYTRLGGMVWRADTKANVPGGASFKDHDTGVSPVFAGGVEYAITPE<br>IATRLEYQWTNNIGDAHTIGTRPDNGMLSLGVSYRFGQGEAAPVVAPAPAPAP<br>EVQTKHFTLKSDVLFTFNKATLKPEGQAALDQLYSQLSNLDPKDGSVVVLGYT<br>DRIGSDAYNQALSERRAQSVVDYLISKGIPADKISARGMGESNPVTGNTCDNV<br>KQRAALIDCLAPDRRVEIEVKGIKDVVTQPQA**
#12	long	Drainage fluid bile duct	O8	MKKTAIAIAVALAGFATVAQAAPKDNTWYTGAKLGWSQYHDTGFIPNNGPTHE<br>NQLGAGAFGGYQVNPYVGFEMGYDWLGRMPYKGDNINGAYKAQGVQLTAKLGY<br>PITDDLDVYTRLGGMVWRADTKANVPGGASFKDHDTGVSPVFAGGVEYAITPE<br>IATRLEYQWTNNIGDANTIGTRPDNGLLSLGVSYRFGQGEAAPVVAPAPAPAP<br>EVQTKHFTLKSDVLFNFNKATLKPEGQAALDQLYSQLSNLDPKDGSVVVLGYT<br>DRIGSDAYNQGLSERRAQSVVDYLISKGIPADKISARGMGESNPVTGNTCDNV<br>KQRAALIDCLAPDRRVEIEVKGIKDVVTQPQA
#13	long	Urine	O25	MKKTAIAIAVALAGFATVAQAAPKDNTWYTGAKLGWSQYHDTGFIPNNGPTHE<br>NQLGAGAFGGYQVNPYVGFEMGYDWLGRMPYKGDNINGAYKAQGVQLTAKLGY<br>PITDDLDIYTRLGGMVWRADTKANVPGGASFKDHDTGVSPVFAGGVEYAITPE<br>IATRLEYQWTNNIGDAHTIGTRPDNGMLSLGVSYRFGQGEAAPVVAPAPAPAP<br>EVQTKHFTLKSDVLFTFNKATLKPEGQAALDQLYSQLSNLDPKDGSVVVLGYT<br>DRIGSDAYNQALSERRAQSVVDYLISKGIPADKISARGMGESNPVTGNTCDNV<br>KQRAALIDCLAPDRRVEIEVKGIKDVVTQPQA
#14	short	Urine	O9	MKKTAIAIAVALAGFATVAQAAPKDNTWYTGAKLGWSQYHDTGFINNNGPTHE<br>NQLGAGAFGGYQVNPYVGFEMGYDWLGRMPYKGSVENGAYKAQGVQLTAKLGY<br>PITDDLDIYTRLGGMVWRADTKSNVYGKNHDTGVSPVFAGGVEYAITPEIATR<br>LEYQWTNNIGDAHTIGTRPDNGMLSLGVSYRFGQGEAAPVVAPAPAPAPEVQT<br>KHFTLKSDVLFNFNKATLKPEGQAALDQLYSQLSNLDPKDGSVVVLGYTDRIG<br>SDAYNQGLSERRAQSVVDYLISKGIPADKISARGMGESNPVTGNTCDNVKQRA<br>ALIDCLAPDRRVEIEVKGIKDVVTQPQA
#15	short	Urine	O1	MKKTAIAIAVALAGFATVAQAAPKDNTWYTGAKLGWSQYHDTGFINNNGPTHE<br>NQLGAGAFGGYQVNPYVGFEMGYDWLGRMPYKGDNINGAYKAQGVQLTAKLGY<br>PITDDLDVYTRLGGMVWRADTKSNVYGKNHDTGVSPVFAGGVEYAITPEIATR<br>LEYQWTNNIGDAHTIGTRPDNGMLSLGVSYRFGQGEAAPVVAPAPAPAPEVQT<br>KHFTLKSDVLFTFNKATLKPEGQAALDQLYSQLSNLDPKDGSVVVLGYTDRIG<br>SDAYNQALSERRAQSVVDYLISKGIPADKISARGMGESNPVTGNTCDNVKQRA<br>ALIDCLAPDRRVEIEVKGIKDVVTQPQA
#16	short	n/a	O153	MKKTAIAIAVALAGFATVAQAAPKDNTWYTGAKLGWSQYHDTGFINNNGPTHE<br>NQLGAGAFGGYQVNPYVGFEMGYDWLGRMPYKGDNINGAYKAQGVQLTAKLGY<br>PITDDLDVYTRLGGMVWRADTKSNVYGKNHDTGVSPVFAGGVEYAITPEIATR<br>LEYQWTNNIGDAHTIGTRPDNGMLSLGVSYRFGQGEAAPVVAPAPAPAPEVQT<br>KHFTLKSDVLFTFNKATLKPEGQAALDQLYSQLSNLDPKDGSVVVLGYTDRIG<br>SDAYNQALSERRAQSVVDYLISKGIPADKISARGMGESNPVTGNTCDNVKQRA<br>ALIDCLAPDRRVEIEVKGIKDVVTQPQA

Clinical strain		OmpA isoform	Sample origin	Sero-group	OmpA protein sequence
	#17	long	Urine	O153	MKKTAIAIAVALAGFATVAQAAPKDNTWYTGAKLGWSQYHDTGFIPNNGPTHE<br>NQLGAGAFGGYQVNPYVGFEMGYDWLGRMPYKGDNINGAYKAQGVQLTAKLGY<br>PITDDLDVYTRLGGMVWRADTKSNVPGGASTKDHDTGVSPVFAGGVEYAITPE<br>IATRLEYQWTNNIGDAHTIGTRPDNGMLSLGVSYRFGQGEAAPVVAPAPAPAP<br>EVQTKHFTLKSDVLFNFNKATLKPEGQAALDQLYSQLSNLDPKDGSVVVLGYT<br>DRIGSDAYNQALSERRAQSVVDYLISKGIPADKISARGMGESNPVTGNTCDNV<br>KQRAALIDCLAPDRRVEIEVKGIKDVVTQPQA
	#18	long	Urine	O25	MKKTAIAIAVALAGFATVAQAAPKDNTWYTGAKLGWSQYHDTGFIPNNGPTHE<br>NQLGAGAFGGYQVNPYVGFEMGYDWLGRMPYKGDNINGAYKAQGVQLTAKLGY<br>PITDDLDIYTRLGGMVWRADTKANVPGGASFKDHDTGVSPVFAGGVEYAITPE<br>IATRLEYQWTNNIGDAHTIGTRPDNGMLSLGVSYRFGQGEAAPVVAPAPAPAP<br>EVQTKHFTLKSDVLFTFNKATLKPEGQAALDQLYSQLSNLDPKDGSVVVLGYT<br>DRIGSDAYNQALSERRAQSVVDYLISKGIPADKISARGMGESNPVTGNTCDNV<br>KQRAALIDCLAPDRRVEIEVKGIKDVVTQPQA
	#19	long	Urine	O83	MKKTAIAIAVALAGFATVAQAAPKDNTWYTGAKLGWSQYHDTGFIPNNGPTHE<br>NQLGAGAFGGYQVNPYVGFEMGYDWLGRMPYKGDNINGAYKAQGVQLTAKLGY<br>PITDDLDIYTRLGGMVWRADTKANVPGGASFKDHDTGVSPVFAGGVEYAITPE<br>IATRLEYQWTNNIGDAHTIGTRPDNGMLSLGVSYRFGQGEAAPVVAPAPAPAP<br>EVQTKHFTLKSDVLFTFNKATLKPEGQAALDQLYSQLSNLDPKDGSVVVLGYT<br>DRIGSDAYNQALSERRAQSVVDYLISKGIPADKISARGMGESNPVTGNTCDNV<br>KQRAALIDCLAPDRRVEIEVKGIKDVVTQPQA
	#20	short	Inguinal swab	O75	MKKTAIAIAVALAGFATVAQAAPKDNTWYTGAKLGWSQYHDTGFINNNGPTHE<br>NQLGAGAFGGYQVNPYVGFEMGYDWLGRMPYKGSVENGAYKAQGVQLTAKLGY<br>PITDDLDVYTRLGGMVWRADTKSNFDGKNHDTGVSPVFAGGVEYAITPEIATR<br>LEYQWTNNIGDAHTIGTRPDNGMLSLGVSYRFGQGEAAPVVAPAPAPAPEVQT<br>KHFTLKSDVLFTFNKATLKPEGQAALDQLYSQLSNLDPKDGSVVVLGYTDRIG<br>SDAYNQALSERRAQSVVDYLISKGIPADKISARGMGESNPVTGNTCDNVKQRA<br>ALIDCLAPDRRVEIEVKGIKDVVTQPQA
	#21	short	Urine	O16	MKKTAIAIAVALAGFATVAQAAPKDNTWYTGAKLGWSQYHDTGFIDNNGPTHE<br>NQLGAGAFGGYQVNPYVGFEMGYDWLGRMPYKGSVENGAYKAQGVQLTAKLGY<br>PITDDLDVYTRLGGMVWRADTKSNVYGKNHDTGVSPVFAGGVEYAITPEIATR<br>LEYQWTNNIGDAHTIGTRPDNGMLSLGVSYRFGQGEAAPVVAPAPAPAPEVQT<br>KHFTLKSDVLFTFNKATLKPEGQAALDQLYSQLSNLDPKDGSVVVLGYTDRIG<br>SDAYNQALSERRAQSVVDYLISKGIPADKISARGMGESNPVTGNTCDNVKQRA<br>ALIDCLAPDRRVEIEVKGIKDVVTQPQA
	#22	long	Urine	O153	MKKTAIAIAVALAGFATVAQAAPKDNTWYTGAKLGWSQYHDTGFINNNGPTHE<br>NQLGAGAFGGYQVNPYVGFEMGYDWLGRMPYKGSVENGAYKAQGVQLTAKLGY<br>PITDDLDVYTRLGGMVWRADTKAHNNVTGESDKNHDTGVSPVFAGGVEWAITP<br>EIATRLEYQWTNNIGDANTIGTRPDNGLLSLGVSYRFGQGEAAPVVAPAPAPA<br>PEVQTKHFTLKSDVLFTFNKATLKPEGQAALDQLYSQLSNLDPKDGSVVVLGY<br>TDRIGSDAYNQGLSERRAQSVVDYLISKGIPADKISARGMGESNPVTGNTCDN<br>VKQRAALIDCLAPDRRVEIEVKGIKDVVTQPQA
	#23	long	n/a	O166	MKKTAIAIAVALAGFATVAQAAPKDNTWYTGAKLGWSQYHDTGFIDNNGPTHE<br>NQLGAGAFGGYQVNPYVGFEMGYDWLGRMPYKGSVENGAYKAQGVQLTAKLGY<br>PITDDLDVYTRLGGMVWRADTKAHNNVTGESEKNHDTGVSPVFAGGVEWAITP<br>EIATRLEYQWTNNIGDAHTIGTRPDNGLLSLGVSYRFGQGEAAPVVAPAPAPA<br>PEVQTKHFTLKSDVLFNFNKATLKPEGQAALDQLYSQLSNLDPKDGSVVVLGY<br>TDRIGSDAYNQGLSERRAQSVVDYLISKGIPADKISARGMGESNPVTGNTCDN<br>VKQRAALIDCLAPDRRVEIEVKGIKDVVTQPQA
	#24	long	Urine	O126	MKKTAIAIAVALAGFATVAQAAPKDNTWYTGAKLGWSQYHDTGFINNNGPTHE<br>NQLGAGAFGGYQVNPYVGFEMGYDWLGRMPYKGDNINGAYKAQGVQLTAKLGY<br>PITDDLDIYTRLGGMVWRADTKANVPGGASFKDHDTGVSPVFAGGVEYAITPE<br>IATRLEYQWTNNIGDAHTIGTRPDNGLLSLGVSYRFGQGEAAPVVAPAPAPAP<br>EVQTKHFTLKSDVLFNFNKATLKPEGQAALDQLYSQLSNLDPKDGSVVVLGYT<br>DRIGSDAYNQGLSERRAQSVVDYLISKGIPADKISARGMGESNPVTGNTCDNV<br>KQRAALIDCLAPDRRVEIEVKGIKDVVTQPQA
	#25	short	Urine	O2	MKKTAIAIAVALAGFATVAQAAPKDNTWYTGAKLGWSQYHDTGFIDNNGPTHE<br>NQLGAGAFGGYQVNPYVGFEMGYDWLGRMPYKGSVENGAYKAQGVQLTAKLGY<br>PITDDLDVYTRLGGMVWRADTKSNFDGKNHDTGVSPVFAGGVEYAITPEIATR<br>LEYQWTNNIGDAHTIGTRPDNGMLSLGVSYRFGQGEAAPVVAPAPAPAPEVQT<br>KHFTLKSDVLFTFNKATLKPEGQAALDQLYSQLSNLDPKDGSVVVLGYTDRIG<br>SDAYNQALSERRAQSVVDYLISKGIPADKISARGMGESNPVTGNTCDNVKQRA<br>ALIDCLAPDRRVEIEVKGIKDVVTQPQA

Clinical strain	OmpA isoform	Sample origin	Sero-group	OmpA protein sequence
#26	short	Urine	O171	MKKTAIAIAVALAGFATVAQAAPKDNTWYTGAKLGWSQYHDTGFINNNGPTHE<br>NQLGAGAFGGYQVNPYVGFEMGYDWLGRMPYKGSVKNGAYKAQGVQLTAKLGY<br>PITDDLDVYTRLGGMVWRADTKSNFDGKNHDTGVSPVFAGGVEYAITPEIATR<br>LEYQWTNNIGDAHTIGTRPDNGMLSLGVSYRFGQGEAAPVVAPAPAPAPEVQT<br>KHFTLKSDVLFTFNKATLKPEGQAALDQLYSQLSNLDPKDGSVVVLGYTDRIG<br>SDAYNQGLSERRAQSVVDYLISKGIPADKISARGMGESNPVTGNTCDNVKQRA<br>ALIDCLAPDRRVEIEVKGIKDVVTQPQA
#27	short	Urine	n/a	MKKTAIAIAVALAGFATVAQAAPKDNTWYTGAKLGWSQYHDTGFINNNGPTHE<br>NQLGAGAFGGYQVNPYVGFEMGYDWLGRMPYKVSVKNGAYKAQGVQLTAKLGY<br>PITDDLDIYTRLGGMVWRADTKSNVYGKNHDTGVSPVFAGGVEYAITPEIATR<br>LEYQWTNNIGDAHTIGTRPDNGMLSLGVSYRFGQGEAAPVVAPAPAPAPEVQT<br>KHFTLKSDVLFNFNKATLKPEGQAALDQLYSQLSNLDPKDGSVVVLGYTDRIG<br>SDAYNQGLSERRAQSVVDYLISKGIPADKISARGMGESNPVTGNTCDNVKQRA<br>ALIDCLAPDRRVEIEVKGIKDVVTQPQA
#28	short	Urine	O9	MKKTAIAIAVALAGFATVAQAAPKDNTWYTGAKLGWSQYHDTGFINNNGPTHE<br>NQLGAGAFGGYQVNPYVGFEMGYDWLGRMPYKGSVKNGAYKAQGVQLTAKLGY<br>PITDDLDIYTRLGGMVWRADTKSNVYGKNHDTGVSPVFAGGVEYAITPEIATR<br>LEYQWTNNIGDAHTIGTRPDNGMLSLGVSYRFGQGEAAPVVAPAPAPAPEVQT<br>KHFTLKSDVLFNFNKATLKPEGQAALDQLYSQLSNLDPKDGSVVVLGYTDRIG<br>SDAYNQGLSERRAQSVVDYLISKGIPADKISARGMGESNPVTGNTCDNVKQRA<br>ALIDCLAPDRRVEIEVKGIKDVVTQPQA
E. coli ATCC 25288 (derived from ATCC 25404)	short	reference	none (K-12)	MKKTAIAIAVALAGFATVAQAAPKDNTWYTGAKLGWSQYHDTGFINNNGPTHE<br>NQLGAGAFGGYQVNPYVGFEMGYDWLGRMPYKGSVENGAYKAQGVQLTAKLGY<br>PITDDLDIYTRLGGMVWRADTKSNVYGKNHDTGVSPVFAGGVEYAITPEIATR<br>LEYQWTNNIGDAHTIGTRPDNGMLSLGVSYRFGQGEAAPVVAPAPAPAPEVQT<br>KHFTLKSDVLFNFNKATLKPEGQAALDQLYSQLSNLDPKDGSVVVLGYTDRIG<br>SDAYNQGLSERRAQSVVDYLISKGIPADKISARGMGESNPVTGNTCDNVKQRA<br>ALIDCLAPDRRVEIEVKGIKDVVTQPQA
E. coli DSM 1103 (ATCC 25922)	long	reference	n/a	MKKTAIAIAVALAGFATVAQAAPKDNTWYTGAKLGWSQYHDTGFIPNNGPTHE<br>NQLGAGAFGGYQVNPYVGFEMGYDWLGRMPYKGDNINGAYKAQGVQLTAKLGY<br>PITDDLDIYTRLGGMVWRADTKANVPGGASFKDHDTGVSPVFAGGVEYAITPE<br>IATRLEYQWTNNIGDAHTIGTRPDNGMLSLGVSYRFGQGEVAPVVAPAPAPAP<br>EVQTKHFTLKSDVLFTFNKATLKPEGQAALDQLYSQLSNLDPKDGSVVVLGYT<br>DRIGSDAYNQALSERRAQSVVDYLISKGIPADKISARGMGESNPVTGNTCDNV<br>KQRAALIDCLAPDRRVEIEVKGIKDVVTQPQA
E. coli DSM 1576 (ATCC 8739)	short	reference	n/a	MKKTAIAIAVALAGFATVAQAAPKDNTWYTGAKLGWSQYHDTGFINNNGPTHE<br>NQLGAGAFGGYQVNPYVGFEMGYDWLGRMPYKGSVENGAYKAQGVQLTAKLGY<br>PITDDLDIYTRLGGMVWRADTKSNVYGKNHDTGVSPVFAGGVEYAITPEIATR<br>LEYQWTNNIGDAHTIGTRPDNGMLSLGVSYRFGQGEAAPVVAPAPAPAPEVQT<br>KHFTLKSDVLFNFNKATLKPEGQAALDQLYSQLSNLDPKDGSVVVLGYTDRIG<br>SDAYNQGLSERRAQSVVDYLISKGIPADKISARGMGESNPVTGNTCDNVKQRA<br>ALIDCLAPDRRVEIEVKGIKDVVTQPQA
E. coli DSM 17076 (ATCC 700728)	short	reference	O157:H7	MKKTAIAIAVALAGFATVAQAAPKDNTWYTGAKLGWSQYHDTGFINNNGPTHE<br>NQLGAGAFGGYQVNPYVGFEMGYDWLGRMPYKGSVENGAYKAQGVQLTAKLGY<br>PITDDLDIYTRLGGMVWRADTKSNVYGKNHDTGVSPVFAGGVEYAITPEIATR<br>LEYQWTNNIGDAHTIGTRPDNGMLSLGVSYRFGQGEAAPVVAPAPAPAPEVQT<br>KHFTLKSDVLFNFNKATLKPEGQAALDQLYSQLSNLDPKDGSVVVLGYTDRIG<br>SDAYNQGLSERRAQSVVDYLISKGIPADKISARGMGESNPVTGNTCDNVKQRA<br>ALIDCLAPDRRVEIEVKGIKDVVTQPQA
E. coli NENT 2540-04	n/a	reference	O157	sequence not available
Alcaligenes faecalis DSM30030		Non-*E. coli* reference		not found
Burkholderia cepacia DSM7288		Non-*E. coli* reference		not found
Citrobacter amalonaticus DSM4593		Non-*E. coli* reference		MKKTAIAIAVALAGFATVAQAAPKDNTWYTGAKLGWSQYHDTGFINNNGPTHE<br>NQLGAGAFGGYQVNPYVGFEMGYDWLGRMPYKGDNINGAYKAQGVQLTAKLGY<br>PITDDLDIYTRLGGMVWRADTKANEPGGASFKDHDTGVSPVFAGGVEYAITPE<br>IATRLEYQWTNNIGDANTIGTRPDNGLLSVGVSYRFGQQEQAAPIVAPAPAPA<br>PEVQTKHFTLKSDVLFNFNKATLKPEGQQALDQMYSQLSNLDPKDGSVVVLGF<br>TDRIGSDAYNQGLSEKRAQSVVDYLISKGIPADKISARGMGESNPVTGNTCDN<br>VKARAALIDCLAPDRRVEIEVKGIKDVVTQPQA

Clinical strain	OmpA isoform	Sample origin	Sero-group	OmpA protein sequence
Citrobacter freundii DSM24397 (ATCC 43864)		Non-*E. coli* reference		MKKTAIAIAVALAGFATVAQAAPKDNTWYTGAKLGWSQYHDIGNNQIDNNGPT<br>HESQLGAGAFGGYQVNPYVGFEMGYDWLGRMPYKGNTENGAFKAQGVQLTAKL<br>GYPITDDLDVYTRLGGMVWRADAKNNQGFKDHDTGVSPVFAGGVEYAITPEIA<br>TRLEYQWTNNIGDANTVGGRPDNGLLSVGVSYRFGQQEEAAPVVVAPAPAPEV<br>QTKHFTLKSDVLFNFNKATLKPEGQQALDQMYSQLSNLDPKDGSVVVLGFTDR<br>IGSDAYNQGLSEKRAQSVVDYLISKGIPSDKISARGMGESNPVTGNTCDNVKA<br>RAALIDCLAPDRRVEIEVKGIKDVVTQPQA
Enterobacter hormaechei DSM12409 (ATCC 49162)		Non-*E. coli* reference		MKKTAIAIAVALAGFATVAQAAPKDNTWYAGGKLGWSQFHDTGWYNSSLNNDG<br>PTHESQLGAGAFGGYQVNPYVGFEMGYDWLGRMPYKGDNVNGAFKAQGVQLTA<br>KLGYPVTDDLDVYTRLGGMVWRADSSNSIAGDDHDTGVSPVFAGGVEWAMTRD<br>IATRLEYQWVNNIGDGATVGVRPDNGMLSVGVSYRFGQQEDAPVVAPAPAPAP<br>EVQTKHFTLKSDVLFNFNKATLKPEGQQALDQLYTQLSNLDPKDGSVVVLGFT<br>DRIGSDAYNQGLSEKRAQSVVDYLVSKGIPANKISPRGMGESNPVTGSTCDNV<br>KPRAALIDCLAPDRRVEIEVKGIKDVVTQPAA
Enterococcus durans DSM20633		Non-*E. coli* reference		not found
Enterococcus faecalis DSM20478		Non-*E. coli* reference		not found
Enterococcus faecalis DSM2570		Non-*E. coli* reference		not found
Enterococcus gallinarum DSM24841		Non-*E. coli* reference		not found
Escherichia fergusonii DSM13698 (ATCC_35469)		Non-*E. coli* reference		MKKTAIAIAVALAGFATVAQAAPKDNTWYTGAKLGWSQYHDTGFIDNNGPTHE<br>NQLGAGAFGGYQVNPYVGFEMGYDWLGRMPYKGSVENGAYKAQGVQLTAKLGY<br>PITDDLDIYTRLGGMVWRADTKAHNNVTGESEKNHDTGVSPVFAGGVEWAITP<br>EIATRLEYQWTNNIGDANTIGTRPDNGLLSLGVSYRFGQGEAAPVVAPAPAPA<br>PEVQTKHFTLKSDVLFNFNKATLKPEGQAALDQLYSQLSNLDPKDGSVVVLGY<br>TDRIGSDAYNQGLSERRAQSVVDYLISKGIPADKISARGMGESNPVTGNTCDN<br>VKQRAALIDCLAPDRRVEIEVKGIKDVVTQPQA
Hafnia alvei DSM30163		Non-*E. coli* reference		MKKTAIALAVALAGFATVAQAAPKDDTWYVGGKLGWSHYYDNSINHFGSTNVR<br>PDQLGGGAFFGYQANPYLGFEMGYDWLGRMEYRGNNNGAFKSQGVQLAAKLSY<br>PIADDLDIYTRLGGMVWRADGSANSETRGRYIDSHDTGVSPLAAIGVEYALNK<br>DWATRLDYQFVSNIGDANETAARPDNTLLSVGVTYRFGQDEAAPVVAPAPAPA<br>PVVETKRFTLKSDVLFNFNKATLKPQGQQALDQMYSQLSNLDPKDGSVVVLGY<br>TDRIGSEQYNQKLSEQRAQSVVDYLVSKGIPADKISARGMGKADPVTGSTCDN<br>VKARAALIDCLAPDRRVEIEVKGIKDVVTQPQA
Klebsiella aerogenes DSM30053 (ATCC 13048)		Non-*E. coli* reference		MKKTAIAIAVALAGFATVAQAAPKDNTWYAGGKLGWSQFHDTGWYNSNLNNNG<br>PTHESQLGAGAFGGYQVNPYLGFEMGYDWLGRMPYKGDNVNGAFKAQGVQLTA<br>KLGYPITDDLDIYTRLGGMVWRADSSNNRIGDNHDTGVSPVFAGGVEWAMTRD<br>IATRLEYQWVNNIGDAGTVGVRPDNGMLSVGVSYRFGQEDNAPVVAPAPAPAP<br>EVTTKTFTLKSDVLFNFNKATLKPEGQQALDQLYTQLSNMDPKDGSAVVLGYT<br>DRIGSEQYNQKLSEKRAQSVVDYLVAKGIPANKISARGMGESDPVTGNTCDNV<br>KARAALIDCLAPDRRVAIEVKGYKDVVTQPQA
Klebsiella pneumoniae DSM681 (ATCC 10031)		Non-*E. coli* reference		MKKTAIAIAVALAGFATVAQAAPKDNTWYAGGKLGWSQYHDTGFYGNGFQNNN<br>GPTRNDQLGAGAFGGYQVNPYLGFEMGYDWLGRMAYKGSVDNGAFKAQGVQLT<br>AKLGYPITDDLDIYTRLGGMVWRADSKGNYASTGVSRSEHDTGVSPVFAGGVE<br>WAVTRDIATRLEYQWVNNIGDAGTVGTRPDNGMLSLGVSYRFGQEDAAPVVAP<br>APAPAPEVATKHFTLKSDVLFNFNKATLKPEGQQALDQLYTQLSNMDPKDGSA<br>VVLGYTDRIGSEAYNQQLSEKRAQSVVDYLVAKGIPAGKISARGMGESNPVTG<br>NTCDNVKARAALIDCLAPDRRVEIEVKGYKEVVTQPAA

Clinical strain	OmpA isoform	Sample origin	Sero-group	OmpA protein sequence
Klebsiella pneumoniae DSM789 (ATCC 4352)		Non-*E. coli* reference		MKKTAIAIAVALAGFATVAQAAPKDNTWYAGGKLGWSQYHDTGFYGNGFQNNN<br>GPTRNDQLGAGAFGGYQVNPYLGFEMGYDWLGRMAYKGSVDNGAFKAQGVQLT<br>AKLGYPITDDLDIYTRLGGMVWRADSKGNYASTGVSRSEHDTGVSPVFAGGVE<br>WAVTRDIATRLEYQWVNNIGDAGTVGTRPDNGMLSLGVSYRFGQEDAAPVVAP<br>APAPAPEVATKHFTLKSDVLFNFNKATLKPEGQQALDQLYTQLSNMDPKDGSA<br>VVLGYTDRIGSEAYNQQLSEKRAQSVVDYLVAKGIPAGKISARGMGESNPVTG<br>NTCDNVKARAALIDCLAPDRRVEIEVKGYKEVVTQPAA
Pseudomonas aeruginosa DSM1117 (ATCC 27853)	OprF	Non-*E. coli* reference		MKLKNTLGVVIGSLVAASAMNAFAQGQNSVEIEAFGKRYFTDSVRNMKNADLY<br>GGSIGYFLTDDVELALSYGEYHDVRGTYETGNKKVHGNLTSLDAIYHFGTPGV<br>GLRPYVSAGLAHQNITNINSDSQGRQQMTMANIGAGLKYYFTENFFAKASLDG<br>QYGLEKRDNGHQGEWMAGLGVGFNFGGSKAAPAPEPVADVCSDSDNDGVCDNV<br>DKCPDTPANVTVDANGCPAVAEVVRVQLDVKFDFDKSKVKENSYADIKNLADF<br>MKQYPSTSTTVEGHTDSVGTDAYNQKLSERRANAVRDVLVNEYGVEGGRVNAV<br>GYGESRPVADNATAEGRAINRRVEAEVEAEAK
Pseudomonas aeruginosa DSM50071 (ATCC 10145)	OprF	Non-*E. coli* reference		MKLKNTLGVVIGSLVAASAMNAFAQGQNSVEIEAFGKRYFTDSVRNMKNADLY<br>GGSIGYFLTDDVELALSYGEYHDVRGTYETGNKKVHGNLTSLDAIYHFGTPGV<br>GLRPYVSAGLAHQNITNINSDSQGRQQMTMANIGAGLKYYFTENFFAKASLDG<br>QYGLEKRDNGHQGEWMAGLGVGFNFGGSKAAPAPEPVADVCSDSDNDGVCDNV<br>DKCPDTPANVTVDANGCPAVAEVVRVQLDVKFDFDKSKVKENSYADIKNLADF<br>MKQYPSTSTTVEGHTDSVGTDAYNQKLSERRANAVRDVLVNEYGVEGGRVNAV<br>GYGESRPVADNATAEGRAINRRVEAEVEAEAK
Salmonella enterica DSM19587 (ATCC 14028)		Non-*E. coli* reference		MKKTAIAIAVALAGFATVAQAAPKDNTWYAGAKLGWSQYHDTGFIHNDGPTHE<br>NQLGAGAFGGYQVNPYVGFEMGYDWLGRMPYKGDNINGAYKAQGVQLTAKLGY<br>PITDDLDVYTRLGGMVWRADTKSNVPGGPSTKDHDTGVSPVFAGGIEYAITPE<br>IATRLEYQWTNNIGDANTIGTRPDNGLLSVGVSYRFGQQEAAPVVAPAPAPAP<br>EVQTKHFTLKSDVLFNFNKSTLKPEGQQALDQLYSQLSNLDPKDGSVVVLGFT<br>DRIGSDAYNQGLSEKRAQSVVDYLISKGIPSDKISARGMGESNPVTGNTCDNV<br>KPRAALIDCLAPDRRVEIEVKGVKDVVTQPQA
Shigella boydii DSM7532 (ATCC 8700)		Non-*E. coli* reference		MKKTAIAIAVALAGFATVAQAAPKDNTWYTGAKLGWSQYHDTGFINNNGPTHE<br>NQLGAGAFGGYQVNPYVGFEMGYDWLGRMPYKGSVENGAYKAQGVQLTAKLGY<br>PITDDLDIYTRLGGMVWRADTKSNVYGKNHDTGVSPVFAGGVEYAITPEIATR<br>LEYQWTNNIGDAHTIGTRPDNGMLSLGVSYRFGQGEAAPVVAPAPAPAPEVQT<br>KHFTLKSDVLFNFNKATLKPEGQAALDQLYSQLSNLDPKDGSVVVLGYTDRIG<br>SDAYNQGLSERRAQSVVDYLISKGIPADKISARGMGESNPVTGNTCDNVKQRA<br>ALIDCLAPDRRVEIEVKGIKDVVTQPQA
Shigella flexneri DSM4782 (ATCC 29903)		Non-*E. coli* reference		MKKTAIAIAVALAGFATVAQAAPKDNTWYTGAKLGWSQYHDTGFIPNNGPTHE<br>NQLGAGAFGGYQVNPYVGFEMGYDWLGRMPYKGDNINGAYKAQGVQLTAKLGY<br>PITDDLDIYTRLGGMVWRADTKANVPGGASFKDHDTGVSPVFAGGVEYAITPE<br>IATRLEYQWTNNIGDANTIGTRPDNGLLSLGVSYRFGQGEAAPVVAPAPAPEV<br>QTKHFTLKSDVLFNFNKATLKPEGQAALDQLYSQLSNLDPKDGSVVVLGYTDR<br>IGSDAYNQGLSERRAQSVVDYLISKGIPADKISARGMGESNPVTGNTCDNVKQ<br>RAALIDCLAPDRRVEIEVKGIKDVVTQPQA
Shigella sonnei DSM5570 (ATCC 29930)		Non-*E. coli* reference		MKKTAIAIAVALAGFATVAQAAPKDNTWYTGAKLGWSQYHDTGFINNNGPTHE<br>NQLGAGAFGGYQVNPYVGFEMGYDWLGRMPYKGSVENGAYKAQGVQLTAKLGY<br>PITDDLDIYTRLGGMVWRADTKSNVYGKNHDTGVSPVFAGGVEYAITPEIATR<br>LEYQWTNNIGDAHTIGTRPDNGMLSLGVSYRFGQGEAAPVVAPAPAPAPEVQT<br>KHFTLKSDVLFNFNKATLKPEGQAALDQLYSQLSNLDPKDGSVVVLGYTDRIG<br>SDAYNQGLSERRAQSVVDYLISKGIPADKISARGMGESNPVTGNTCDNVKQRA<br>ALIDCLAPDRRVEIEVKGIKDVVTQPQA
Staphylo-coccus aureus DSM799 (ATCC 6538)		Non-*E. coli* reference		not found
Vibrio cholerae NCTC8021 (ATCC 14035)		Non-*E. coli* reference		MKKLAAIISATLLFASSAAVAEVYVGGKVGKSWLDDACLAGQSCEDDDQVVGA<br>FLGYQANKWLSLEAGYDYLGKFTAAGLNDEKVQAVTLAPKLSIPLTEGIALYG<br>KVGGAYVDYGSKDDYSYLGAAGLEFNTNHNVTMRLEYQNLTDINNDIVRARAE<br>TATLGIAYKFGGSEEPAPVVEQRPAEPAPVAAPVEKVAVTKTFTFQHLDSSTF<br>ATASAELKPATVQKLDKIVGYLNQYPQAKVEVVGHTDSTGSEAYNQKLSERRA<br>QAVAKALEAQGIDASRISAKGLGESSPIASNATAEGREKNRRVELVIPEFQYQ<br>VTE
Alcaligenes faecalis DSM30030		Non-*E. coli* reference		not found

Supplementary Table 2 | Primary data of flycode analysis of nanobody selections against OmpA-short. Shown are 13 selected nanobodies with their deep sequencing identifier, number of maximally detectable flycodes (# FC), number of peptides identified by mass spectrometry (# Peptides) and the summed MS1 intensities detected for the indicated strains.

Nb #	Identifier	# FC	# Peptides	MC1061 wt	MC1061 Δ*ompA*	CS #1	CS #2	CS #3	CS #4	CS #5	CS #6
**1**	NB00648	17	6	2,50E+07	1,84E+05	4,26E+07	8,64E+07	9,78E+07	1,56E+08	3,82E+05	3,27E+05
**2**	NB00564	12	6	4,25E+07	3,49E+05	6,46E+07	3,33E+07	6,79E+07	5,93E+07	6,91E+05	2,52E+05
**3**	NB00357	27	14	1,53E+08	1,70E+06	2,63E+08	3,75E+08	3,30E+08	6,19E+08	2,88E+06	2,28E+06
**4**	NB00886	9	5	1,03E+08	1,33E+06	1,01E+08	1,05E+08	1,15E+08	1,84E+08	2,53E+06	1,75E+06
**5**	NB00323	32	10	1,46E+08	3,00E+06	2,05E+08	4,09E+08	3,34E+08	6,97E+08	8,38E+06	3,66E+06
**6**	NB00201	24	11	4,85E+07	1,17E+06	6,94E+07	6,34E+07	9,11E+07	1,06E+08	2,14E+06	1,55E+06
**7**	NB00182	79	9	2,34E+06	1,14E+05	1,22E+06	1,28E+06	3,33E+05	2,96E+05	3,48E+05	2,72E+05
**8**	NB00047	36	16	1,34E+08	7,75E+06	1,97E+08	2,35E+08	2,93E+08	2,77E+08	1,15E+07	8,49E+06
**9**	NB00635	33	12	1,45E+07	9,89E+05	1,57E+07	1,77E+07	1,52E+07	4,00E+07	2,53E+06	9,75E+05
**10**	NB00168	54	6	2,07E+07	1,72E+06	2,07E+07	1,22E+07	1,22E+07	2,05E+07	2,97E+06	2,11E+06
**11**	NB00408	14	6	1,70E+07	1,53E+06	1,86E+07	9,84E+06	1,62E+07	1,52E+07	2,25E+06	2,19E+06
**12**	NB00020	134	19	8,14E+06	9,40E+05	1,48E+07	3,29E+06	3,94E+06	2,86E+06	1,30E+07	3,99E+06
**13**	NB00345	49	9	1,13E+07	1,33E+06	3,12E+07	6,13E+06	9,98E+06	1,13E+07	1,94E+07	7,51E+06

Supplementary Table 3 | Binder ranking of nanobodies targeting OmpA-short based on primary flycode data shown in Supplementary Table 2. Shown are 13 selected nanobodies with their deep sequencing identifier, and ratios of summed MS1 intensities of indicated strain pairs. Data for the isogenic lab strain pair (MC1061 wt versus MC1061 *ΔompA*) are shaded in blue. For the clinical strains, no isogenic pairs were available and summed MS1 intensities were compared to MC1061 *ΔompA*. Corresponding values are shaded grey. Nb01 was the top binder regarding the MC1061 wt / MC1061 *ΔompA* ratio and exhibited strong binding in clinical strains expressing OmpA-short, i.e. CS#1 – CS#4.

Nb #	Identifier	MC1061 wt/Δ*ompA*	CS #1/MC1061 Δ*ompA*	CS #2/MC1061 Δ*ompA*	CS #3/MC1061 Δ*ompA*	CS #4/MC1061 Δ*ompA*	CS #5/MC1061 Δ*ompA*	CS #6/MC1061 Δ*ompA*
**1**	NB00648	135,75	231,44	470,01	531,85	850,40	2,08	1,78
**2**	NB00564	121,80	185,15	95,29	194,58	169,76	1,98	0,72
**3**	NB00357	90,13	154,74	221,00	194,25	364,42	1,69	1,34
**4**	NB00886	77,20	75,74	78,45	86,32	138,33	1,90	1,31
**5**	NB00323	48,77	68,17	136,35	111,33	232,14	2,79	1,22
**6**	NB00201	41,30	59,11	53,97	77,56	90,25	1,82	1,32
**7**	NB00182	20,46	10,64	11,17	2,91	2,59	3,04	2,38
**8**	NB00047	17,33	25,38	30,27	37,74	35,72	1,48	1,09
**9**	NB00635	14,67	15,92	17,88	15,42	40,43	2,56	0,99
**10**	NB00168	12,08	12,07	7,09	7,10	11,93	1,73	1,23
**11**	NB00408	11,09	12,15	6,42	10,61	9,91	1,47	1,43
**12**	NB00020	8,66	15,78	3,50	4,19	3,04	13,81	4,25
**13**	NB00345	8,49	23,43	4,60	7,49	8,51	14,56	5,64

Supplementary Table 4 | Primary data of flycode analysis of nanobody selections against OmpA-long. Shown are 5 selected nanobodies with their deep sequencing identifier, number of maximally detectable flycodes (# FC), number of peptides identified by mass spectrometry (# Peptides) and the summed MS1 intensities detected for the indicated strains.

Nb #	Identifier	# FC	# Peptides	MC1061 Δ*ompA*	MC1061 Δ*ompA ::ompA_short*	MC1061 Δ*ompA::ompA_long*	CS #8	CS #11
**38**	NB0011	33	6	1,67E+06	1,28E+06	5,51E+08	5,62E+08	6,46E+08
**39**	NB0060	24	7	5,13E+06	5,37E+06	2,38E+09	4,10E+09	4,18E+09
**40**	NB0065	94	20	1,38E+06	1,75E+06	3,22E+08	4,10E+08	4,73E+08
**41**	NB0076	44	10	3,89E+05	6,31E+04	1,63E+08	6,62E+07	7,37E+07
**42**	NB0161	41	9	2,77E+05	3,12E+06	9,24E+07	4,42E+07	7,40E+07

Supplementary Table 5 | Binder ranking of nanobodies targeting OmpA-long based on primary flycode data shown in Supplementary Table 4. Shown are 5 selected nanobodies with their deep sequencing identifier, and ratios of summed MS1 intensities of indicated strain pairs. Data for the isogenic lab strain pair (MC1061 *ΔompA* expressing either *ompA-long* or *ompA-short* from an expression plasmid) are shaded in blue. For the clinical strains producing OmpA-long (CS#8 and CS#11), no isogenic pairs were available and summed MS1 intensities were compared to MC1061 *ΔompA*. Corresponding values are shaded grey. Nb39 was chosen as the top binder because it exhibited the strongest binding signals in clinical strains.

Nb #	Identifier	MC1061 Δ*ompA::ompA_long* / MC1061 Δ*ompA::ompA_short*	MC1061 Δ*ompA::ompA_long* / MC1061 Δ*ompA*	CS #8/ MC1061 Δ*ompA*	CS #11/ MC1061 Δ*ompA*
**38**	NB0011	431,25	329,30	335,68	386,13
**39**	NB0060	443,49	464,94	800,24	814,83
**40**	NB0065	184,25	233,65	297,58	342,88
**41**	NB0076	2591,87	419,95	169,96	189,24
**42**	NB0161	29,60	333,07	159,17	266,81

Supplementary Table 6 | Protein sequences of selected nanobodies targeting OmpA-short (Nb01-Nb13), OmpF (Nb18-Nb25), OmpA_long (Nb38-Nb42). CDRs are underlined.

Nb	Identifier	Sequence
**Nb01**	NB00648	SQVQLVESGGGLVQPGGSLRLSCVVSGTGFTFSKSPMSWARQAPGKEREWVSAIFADSSTYYSDSVRGRFTISRD<br>NAKNTVYLQMNNVKPEDTAVYYCGHRRLGKTTYDYRGKGTRVTVS
**Nb02**	NB00564	SQMQFVESGGGLVQPGGSLRLSCVVSGTGFTFSKSPMSWARQAPGKEREWVSAIFADSSTYYADSVKGRFTISRD<br>NAKNTVYLQMNDVQPEDSAVYYCGHRRLGKRTYDYRGQGTPVTVS
**Nb03**	NB00357	SQGQLVESGGGLVPPGGSLRLSCAVSGTGFTFSKSPMSWARQAPGKEREWVSAIFADSSTYYSDSVRGRFTISRD<br>NAKNTVYLQMNNVKPEDTAVYYCGHRRLGKTTYDYRGKGTRVTVS
**Nb04**	NB00886	SQRQLVESGGGLVQPGGSLRLSCVVSGTGFTFSKSPMSWARQAPGKEREWVSAIFADSSTYYADSVKGRFTISRD<br>NAKNTVYLQMNDVQPEDSAVYYCGHRRLGKRTYDYRGKGTPVTVS
**Nb05**	NB00323	SQRQLVESGGGLVQPGGSLRLSCVVSGSGFTFSKSPMSWARQAPGKEREWVSAIFADSSTYYADSVKGRFTISRD<br>NAKNTVYLQMNSVKPEDTAVYYCGHRRLGKTTYDYRGQGTRVTVS
**Nb06**	NB00201	SQVQLVESGGGLVQPGGSLRLSCVVSGTGFTFSKSPMSWARQAPGKEREWVSAIFADSSTYYSDSVRGRFTISRD<br>NAKNTVYLEMNNVKPEDTAVYYCGHRRLGKTTYDYRGQGTRVTVS
**Nb07**	NB00182	SQGQLLESGGGLVQPGGSLRLSCAASGLAFSSYASGWYRQAPGKERELVAAMTARGGFTNYADSVKGRFTISRDN<br>GKNTVYLQMNSLKPEDTAVYYCNADPRRYGSQVYWGQGTPVTVS
**Nb08**	NB00047	SQMQLVESGGGLVQPGGSLRLSCVVSGSGFTFSKSPMSWARQAPGKEREWVSAIFADSSTYYADSVKGRFTISRD<br>NAKNTVYLQMNSVKPEDTAVYYCGYRRLGKTTYDYRGQGTPVTVS
**Nb09**	NB00635	SQLQFVESGGGMVQPGGSLRLSCVASGFRFSSVALSWYRQAPGRDRELVALITNDHKSRYGDFVKDRFTISRDNN<br>KNTVYLQMNNLKPEDTAVYSCGVSLLGSNYGRYWGKGTRVTVS
**Nb10**	NB00168	SQGQLVESGGGLVQPGGSLRLSCTASGFTFSSYAMTWHRQAPGKERELVALITNDARTRYGDFVKGRFTISRDNA<br>KNTIYLQMNTLAPEDTALYYCGVSLLGRNYGQHWGKGTRVTVS
**Nb11**	NB00408	SQGQLVESGGGLVQAGGSLRLSCAASGRTFNPYAMGWFRQAPGKEREFVAAIRWSGGSINYADSVKGRFTISRDN<br>AKNTVYLQMNSLKPEDTAVYYCNAAKSLGVWAREYDDWGKGTPVTVS
**Nb12**	NB00020	SQRQLVESGGGLVQAGGSLRLSCAASGSTSSIDTMSWYRQTTGNEREMVASITKGGGRPYYDFSVKGRFTISRDN<br>DKNTMSMQMNSLKPEDTAVYYCNARDSNSGFYYWGKGTPVTVS
**Nb13**	NB00345	SQGQFVESGGALVQPGGSLRLSCAASGFTFSSALMRWYRQAAGKERELVAYVNNKDLYTFYVDSVKGRFTISRDN<br>AKNTVYLQMNSLKPEDTAVYYCNAASTPNTWGQGTRVTVS
**Nb18**	NB00242	SQRQLVESGGGLVQAGGSLTLSCSATGTVPRIDAMGWYRRSTGKKREQVASVGRGGRTNYSDSAKGRFTISRNGN<br>TVTLQMTSLKPEDTDVYFCNALKYGRNADYDDYWGRGTRVTVS
**Nb19**	NB00168	SQVQLVESGGGLVQPGGSLTLSCSAAGTVPRIDAMGWYRRSTGKKREQVASVGRGGRTNYSDSAKGRFTISRNGN<br>TVTLQMTSLKPEDTDVYFCNALKYGRNADYDDYWGQGTPVTVS
**Nb20**	NB00051	SQVQLVESGGGLVQAGGSLTLSCSAAGTVPRIDAMGWYRRSTGKKREQVASVGRGGRTNYSDSAKGRFTISRNGN<br>TVTLQMTSLKPEDTDVYFCNALKYGRNADYDDYWGQGTRVTVS
**Nb21**	NB00204	SQMQLVESGGGLAQAGESLTLSCTVTGSGIRIDAMGWYRRGTGKKREQVASIGRGGRTNYADSAKGRFTISRNGN<br>TMSLRMNNLKPEDTDLYWCNGLRYLRNADYDDYWGQGTRVTVS
**Nb22**	NB00099	SQRQLVESGGGLVEAGGSLLLSCTASGRAPRIDAMGWYRQSTGKKRVQVASIGRQGRTNYSDSAKGRFIISRNGN<br>TVNLQMNNLKPEDTDVYYCNALTYRRNADYDDYWGKGTPVTVS
**Nb23**	NB00140	SQVQLVESGGGLVQAGGSLTLSCSASGRPPRIDAMGWYRQGTGKKREQVASIGRNGRTNYADSVKGRFTISRTGN<br>TVTLQMTSLKPEDTDVYFCNALKYGRNADYDDYWGQGTRVTVS
**Nb24**	NB00329	SQRQLVESGGGLVRAGESLNLSCVASGRSTGSISAMGWMRQGPTKKRELVAAITPKGRANYADSMKDRATISNGA<br>RNSVTLQINNMKPSDTNTYWCYALVRIGRDTDDYWGKGTRVTVS
**Nb25**	NB00379	SQLQLVESGGGLVQSGGSLRLSCTASGSMSGVFAMGWQRQGTGKKRELVAAITKEGRPNYAASVQGRFTISRDGA<br>NTVSLQMNNLKPEDTSVYYCYALRRSGRDTDDYWGKGTRVTVS

**Nb38**	NB0011	SQRQLVESGGGTVQTGGSLRLSCVPNGSIFNFNLMGWYRQSSGQQRELVATLTRDGSENYAEFVKGRFTISRDSG KNTMYLQMTDVKPSDTAVYICHANYRIGRNDLPVWGKGTRVTVS
**Nb39**	NB0060	SQRQLVESGGGLVHTGGSLKLSCVPNGSIFNFNPMGWYRQVSGQQRELVATLTRDGVENYASSVKGRFTISRDSA KNTLYLQMTDVKPGDAAVYICHANYRIGRNDLPVWGKGTPVTVS
**Nb40**	NB0065	SQRQLVESGGGLVQPGGSLRLSCVPNGSIFNFNIMGWYRQNAGNQRELVATMTRDGSASYSDSVKGRFTISRDVD KNTIYLQLDSVKPEDTAVYICHANYRIGRNDLPVWGRGTRVTVS
**Nb41**	NB0076	SQRQLVESGGGSMQPGESLTLSCEASDNILQFGNMGWYRQSPGTQRELVARIHKRGDSDYGDFAKGRFTISRDTV KNKVYLQMTDLKPEDSANYICNGQYVIGRNRLDVWGQGTPVTVS
**Nb42**	NB0161	SQLQLVESGGGLVQPGGSLRLSCVPNGGIFNFNIMGWYRQNAGNQRELVATMTRDGSASYSDSVKGRFTISRDVD KNTIYLQMDSVEPEDTAVYICHANYRIGRNDLPVWGQGTPVTVS

Supplementary Table 7 | X-ray crystallography data collection and refinement statistics.

	OmpA-short<br>Nb01 (PDB: 9FZC)	OmpA-long<br>Nb39 (PDB: 9FZD)
**Data collection**[#]		
Space group	$P2_1$	I222
Cell dimensions		
a, *b*, *c* (Å)	56.05, 55.30, 107.81	52.40, 52.57, 256.75
α, β, γ (°)	90.00, 91.09, 90.00	90.00, 90.00, 90.00
Resolution (Å)	2.28 (2.36-2.28)[*]	2.30 (2.382- 2.30)
R_{meas}	0.16 (0.91)	0.07 (0.93
$I / \sigma I$	11.36 (2.14)	22.03 (3.17)
Completeness (%)	99.9 (99.9)	99.57 (99.94)
Redundancy	6.8 (6.7)	13.2 (13.5)
$CC_{1/2}$ (%)	99.7 (71.5)	99.9 (87.9)
**Refinement**		
Resolution range (Å)	108-2.28 (2.36-2.28)[*]	44.8-2.30 (2.382- 2.30)
No. unique reflections	30638 (3039)	16264 (1601)
R_{work} / R_{free}	20.9/23.6	25.53/28.21
No. atoms		
Protein	4’898	2’255
Ligand/ion	444	0
Water	276	48
B-factors		
Protein	30.5	75.32
Ligand/ion	37.6	
Water	33.1	58.0
R.m.s. deviations		
Bond lengths (Å)	0.002	0.013
Bond angles (°)	0.60	1.80
Ramachandran favored (%)	97.9	96.6
Ramachandran allowed (%)	2.1	3.4
Ramachandran outliers (%)	0	0

[*]Values in parentheses are for highest-resolution shell.

[#]All datasets have been anisotropy corrected subsequently.

Supplementary Table 8 | Specificity analysis of OmpA nanobodies. Overview of nanobody specificities for different *E. coli* and non-*E. coli* bacterial strains as determined by flow cytometry analysis. For evaluation of Nb01 and Nb39 specificities, the ratio of nanobody event counts with respect to the SYBR Green event count was calculated. Bacterial strains with ratios higher than 50 % with respect to the SYBR Green reference are considered detected and bacterial strains with ratios below 1 % are considered not detected. Exemplary dot plots for *E. coli* CS#2 and CS#6 are shown in Supplementary Fig. 7.

**Strain**	**SYBR.pos event count [intact bacteria/mL]**	**Nb01 event count [intact bacteria/mL]**	**Nb39 event count [intact bacteria/mL]**	**Nb01 / ref. [%]**	**Nb39 / ref. [%]**	**Nb01 detection**	**Nb39 detection**
E. coli CS #1	64,020	69,200	0	108.1	0.0	Yes	No
E. coli CS #2	128,280	131,810	30	102.8	0.0	Yes	No
E. coli CS #3	116,190	123,140	30	106.0	0.0	Yes	No
E. coli CS #4	132,910	135,040	0	101.6	0.0	Yes	No
E. coli CS #5	110,130	20	105,200	0.0	95.5	No	Yes
E. coli CS #6	110,340	0	102,230	0.0	92.6	No	Yes
E. coli CS #7	128,640	20	118,190	0.0	91.9	No	Yes
E. coli CS #8	112,950	20	102,510	0.0	90.8	No	Yes
E. coli CS #9	122,770	0	117,630	0.0	95.8	No	Yes
E. coli CS #10	122,640	20	110,250	0.0	89.9	No	Yes
E. coli CS #11	241,860	0	235,900	0.0	97.5	No	Yes
E. coli CS #12	148,050	40	156,970	0.0	106.0	No	Yes
E. coli CS #13	154,180	20	163,150	0.0	105.8	No	Yes
E. coli CS #14	152,400	153,530	330	100.7	0.2	Yes	No
E. coli CS #15	142,390	20	230	0.0	0.2	No	No
E. coli CS #16	272,930	30	290	0.0	0.1	No	No
E. coli CS #17	78,430	30	61,480	0.0	78.4	No	Yes
E. coli CS #18	302,980	30	331,600	0.0	109.4	No	Yes
E. coli CS #19	411,920	10	523,430	0.0	127.1	No	Yes
E. coli CS #20	476,080	20	320	0.0	0.1	No	No
E. coli CS #21	269,340	298,460	250	110.8	0.1	Yes	No
E. coli CS #22	129,160	120	220	0.1	0.2	No	No
E. coli CS #23	135,790	20	350	0.0	0.3	No	No
E. coli CS #24	118,380	30	129,000	0.0	109.0	No	Yes
E. coli CS #25	153,630	40	120	0.0	0.1	No	No
E. coli CS #26	72,240	30	130	0.0	0.2	No	No
E. coli CS #27	144,500	20	140	0.0	0.1	No	No
E. coli CS #28	233,660	10	80	0.0	0.0	No	No
E. coli ATCC 25288	140,250	173,700	160	123.9	0.1	Yes	No
E. coli DSM 1103	303,930	0	286,280	0.0	94.2	No	Yes
E. coli DSM 1576	336,750	366,410	170	108.8	0.1	Yes	No
E. coli DSM 17076	68,000	67,620	0	99	0	Yes	No
E. coli NENT 2540-04	86,920	90,780	0	104	0	Yes	No
E. coli ATCC 29552	157,050	167,480	330	106.6	0.2	No	Yes
E. coli ATCC BAA-2190	182,090	158,020	0	86.8	0.0	Yes	No
E. coli ATCC BAA-2212	154,390	10	173,080	0.0	112.1	No	Yes
E. coli ATCC BAA-2214	193,810	60	223,960	0.0	115.6	Yes	No
E. coli ATCC BAA-2216	143,880	100	156,770	0.1	109.0	Yes	No
E. coli ATCC BAA-2649	46,910	49,570	170	105.7	0.4	No	Yes
Shigella boydii DSM 7532	148,430	185,740	80	125.	0.	Yes	No
Shigella flexneri DSM 4782	210,530	0	211,830	0.	100.	No	Yes
Shigella sonnei DSM 5570	127,610	147,220	80	115.	0.	Yes	No
Alcaligenes faecalis DSM30030	3,190	30	20	0.9	0.6	No	No
Burkholderia cepacia DSM7288	312,900	50	150	0.0	0.0	No	No
Citrobacter amalonaticus DSM4593	378,000	10	110	0.0	0.0	No	No
Citrobacter freundii DSM24397	154,850	20	0	0.0	0.0	No	No
Enterobacter hormaechei DSM12409	415,110	0	30	0.0	0.0	No	No
Enterococcus durans DSM20633	33,900	10	0	0.0	0.0	No	No
Enterococcus faecalis DSM20478	80,090	0	0	0.0	0.0	No	No
Enterococcus faecalis DSM2570	76,220	0	0	0.0	0.0	No	No
Enterococcus gallinarum DSM24841	80,070	0	20	0.0	0.0	No	No
Escherichia fergusonii DSM13698	119,250	0	0	0.0	0.0	No	No
Hafnia alvei DSM30163	467,310	0	0	0.0	0.0	No	No
Klebsiella aerogenes DSM130053	711,300	90	310	0.0	0.0	No	No
Klebsiella pneumoniae DSM681	563,590	0	0	0.0	0.0	No	No
Klebsiella pneumoniae DSM789	178,730	20	0	0.0	0.0	No	No
Pseudomonas aeruginosa DSM1117	211,630	10	40	0.0	0.0	No	No
Pseudomonas aeruginosa DSM50071	1,116,250	0	80	0.0	0.0	No	No
Staphylococcus aureus DSM799	115,070	250	250	0.2	0.2	No	No
Vibrio cholerae NCTC8021	142,080	120	140	0.1	0.1	No	No

Supplementary Table 9 | Primary data of flycode analysis of nanobody selections against OmpF. Shown are 8 selected nanobodies with their deep sequencing identifier, number of maximally detectable flycodes (# FC), number of peptides identified by mass spectrometry (# Peptides) and the summed MS1 intensities detected for the indicated strains.

Nb #	Identifier	# FC	# Peptides	MC1061 wt	MC1061 Δ*ompF*	CS #1	CS #2	CS #5	CS #6	CS #8	CS #10
**18**	NB00242	22	9	8,58E+07	2,76E+06	1,46E+07	2,11E+07	1,31E+07	3,69E+07	1,10E+07	9,22E+06
**19**	NB00168	14	5	9,96E+06	3,48E+05	3,15E+06	1,90E+06	1,56E+06	6,11E+06	2,32E+06	2,09E+06
**20**	NB00051	52	11	1,05E+08	3,97E+06	3,60E+07	2,85E+07	2,09E+07	6,41E+07	2,28E+07	2,40E+07
**21**	NB00204	47	13	2,97E+07	1,13E+06	8,81E+06	7,45E+06	4,93E+06	1,69E+07	6,81E+06	5,66E+06
**22**	NB00099	33	12	1,51E+07	1,13E+06	3,51E+06	5,29E+06	3,58E+06	6,78E+06	4,04E+06	2,86E+06
**23**	NB00140	23	6	8,15E+06	6,54E+05	2,48E+06	2,19E+06	1,83E+06	4,64E+06	2,36E+06	2,07E+06
**24**	NB00329	23	11	1,02E+08	8,71E+06	2,34E+07	6,21E+07	2,94E+07	3,14E+07	2,51E+07	1,64E+07
**25**	NB00379	27	12	4,04E+07	3,89E+06	1,19E+07	1,87E+07	9,84E+06	7,04E+06	8,00E+06	1,06E+07

Supplementary Table 10 | Binder ranking of nanobodies targeting OmpF based on primary flycode data shown in Supplementary Table 9. Shown are 8 selected nanobodies with their deep sequencing identifier, and ratios of summed MS1 intensities of indicated strain pairs. Data for the isogenic lab strain pair (MC1061 wt versus MC1061 Δ*ompF*) are shaded in blue. For the clinical strains, no isogenic pairs were available and summed MS1 intensities were compared to MC1061 Δ*ompF*. Corresponding values are shaded grey. Nb18 was chosen as the top binder because of the highest MC1061 wt / MC1061 Δ*ompA* ratio.

Nb #	Identifier	MC1061 wt/Δ*ompF*	CS #1/MC1061 Δ*ompF*	CS #2/MC1061 Δ*ompF*	CS #5/MC1061 Δ*ompF*	CS #6/MC1061 Δ*ompF*	CS #8/MC1061 Δ*ompF*	CS #10/MC1061 Δ*ompF*
**18**	NB00242	31,08	5,28	7,66	4,73	13,37	3,98	3,34
**19**	NB00168	28,64	9,06	5,46	4,49	17,58	6,66	6,01
**20**	NB00051	26,54	9,07	7,18	5,27	16,15	5,76	6,05
**21**	NB00204	26,22	7,78	6,58	4,36	14,89	6,01	5,00
**22**	NB00099	13,36	3,10	4,67	3,17	5,99	3,57	2,53
**23**	NB00140	12,47	3,80	3,35	2,80	7,10	3,61	3,17
**24**	NB00329	11,75	2,68	7,13	3,37	3,61	2,88	1,89
**25**	NB00379	10,38	3,05	4,80	2,53	1,81	2,05	2,71

Supplementary Table 11 | Primers used in this study.

Primer Name	Primer Sequence	Function
**ompA_FW**	atatatGCTCTTCtAGTgctccgaaagataacacctggtacact	Amplification of *ompA* genes
**ompA_RV**	tatataGCTCTTCaTGCTgcctgcggctgagttacaacgtcttt	Amplification of *ompA* genes
**ompF_FW**	atatatGCTCTTCtAGTgcagaaatctataacaaagatggcaacaaagtag	Amplification of *ompF* gene
**ompF_RV**	tatataGCTCTTCaTGCgaactggtaaacgatacccacagcaac	Amplification of *ompF* gene
**OmpA_TMD_RV**	atatatGCTCTTCATGCGAAACGGTAGGAAACACCCAGGC	TMD amplification
**Kan_cassette_FW**	tatataGCTCTTCaTGGCGACTTCTACTGTAACTGGCGGT	Amplification of Kan cassette
**Kan_cassette_RV**	tatataGCTCTTCaCACCGTAAGAGAAGTCCAGAGCAACG	Amplification of Kan cassette
**ompA_EC_FX_5_FW**	tatataGCTCTTCaAGTCCATTGTTACAGCAACTCGGTCAGC	Amplification of upstream (5') fragment for deletion of *ompA* in *E. coli* MC1061
**ompA_EC_FX_5_RV**	tatataGCTCTTCaCCACTTACAAGTGTGAACTCCGTCAGGCATATG	Amplification of upstream (5') fragment for deletion of *ompA* in *E. coli* MC1061
**ompA_EC_FX_3_FW**	tatataGCTCTTCaGTGCAACGTGAAACAGCGTGCTGC	Amplification of downstream (3') fragment for deletion of *ompA* in *E. coli* MC1061
**ompA_EC_FX_3_RV**	tatataGCTCTTCaTGCGGAAGTATCTGGTTAAGAAGCATCGCG	Amplification of downstream (3') fragment for deletion of *ompA* in *E. coli* MC1061
**ompA_EC_DKO_5_FW**	GGCAACTCTGGTTAACACCGCAAC	Amplification of Kan cassette including flanking regions required for homologous recombination
**ompA_EC_DKO_3_RV**	CCAGGAAGCCGTCGATGTGTTG	Amplification of Kan cassette including flanking regions required for homologous recombination
**ompF_EC_FX_5_FW**	tatataGCTCTTCaAGTCGTGCGAGCACGTTTGTCATTG	Amplification of upstream (5') fragment for deletion of *ompF* in *E. coli* MC1061
**ompF_EC_FX_5_RV**	tatataGCTCTTCaCCAGTTGCTGTGGGTATCGTTTACCAGTTC	Amplification of upstream (5') fragment for deletion of *ompF* in *E. coli* MC1061
**ompF_EC_FX_3_FW**	tatataGCTCTTCaGTGCGACCTGGCACAAAATCTTCTTTCAGAC	Amplification of downstream (3') fragment for deletion of *ompF* in *E. coli* MC1061
**ompF_EC_FX_3_RV**	tatataGCTCTTCaTGCGTTCTATATGCGCCTTAACGAAGAC	Amplification of downstream (3') fragment for deletion of *ompF* in *E. coli* MC1061
**ompF_EC_DKO_5_FW**	CGCTACCTTTACCAAACAGCAGTTCC	Amplification of Kan cassette including flanking regions required for homologous recombination
**ompF_EC_DKO_3_RV**	CGTTGCGGCTATGGACGTTCTG	Amplification of Kan cassette including flanking regions required for homologous recombination
**OmpF_S75C_FW**	CTCAAATCAATTgCGATCTGACCGG	Quickchange-primer to introduce cysteine for site-specific biotinylation
**OmpF_S75C_RV**	CCGGTCAGATCGcAATTGATTTGAG	Quickchange-primer to introduce cysteine for site-specific biotinylation
**pSBinit_Cys_FW**	GCAGGAAGAGCTtgtGGCGAACAAAAAC	Quickchange-primer to introduce cysteine between C-terminal SapI recognition site and the Myc-tag of nanobodies
**pSBinit_Cys_RV**	GTTTTTGTTCGCCacaAGCTCTTCCTGC	Quickchange-primer to introduce cysteine between C-terminal SapI recognition site and the Myc-tag of nanobodies

a

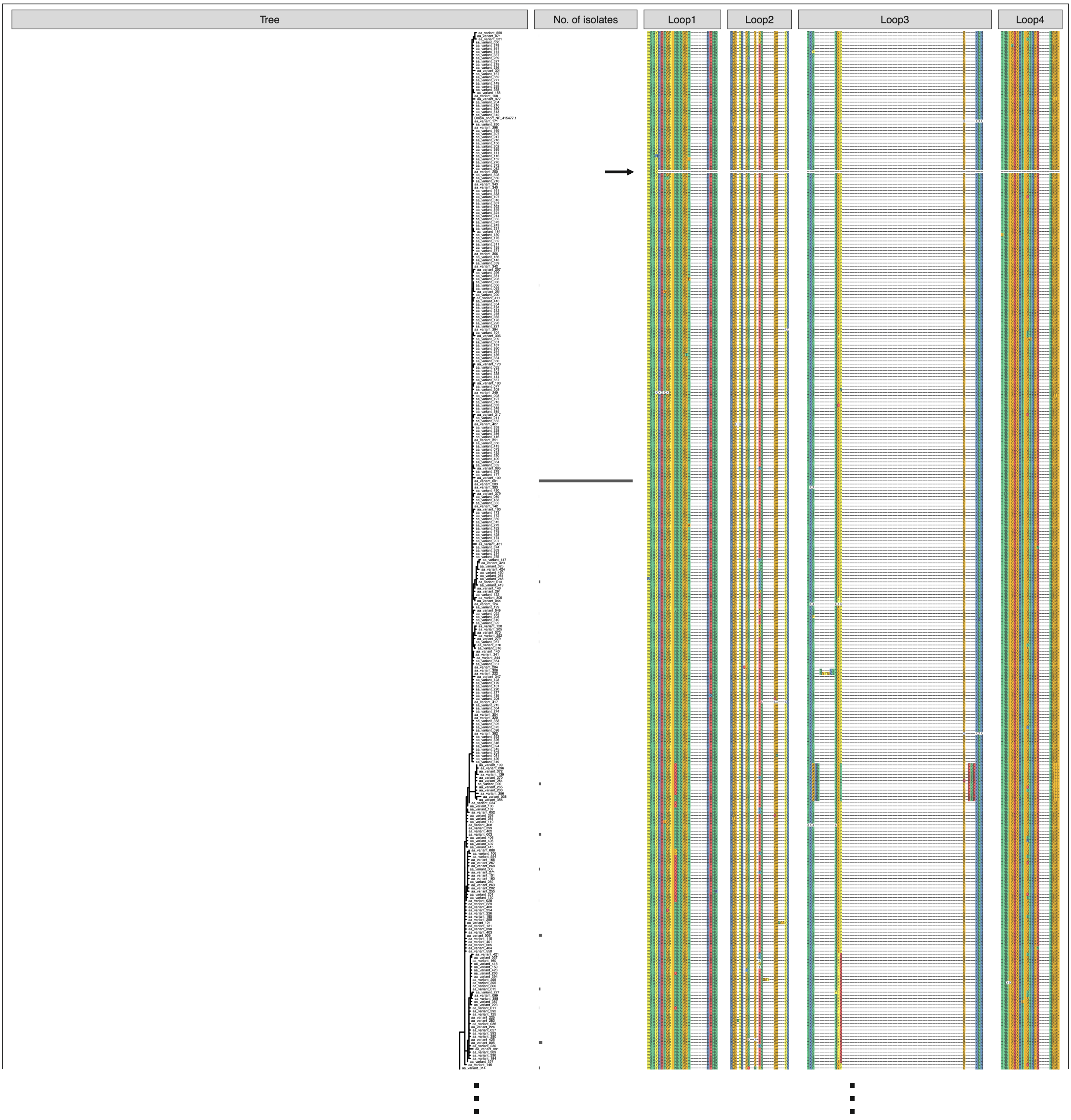

b

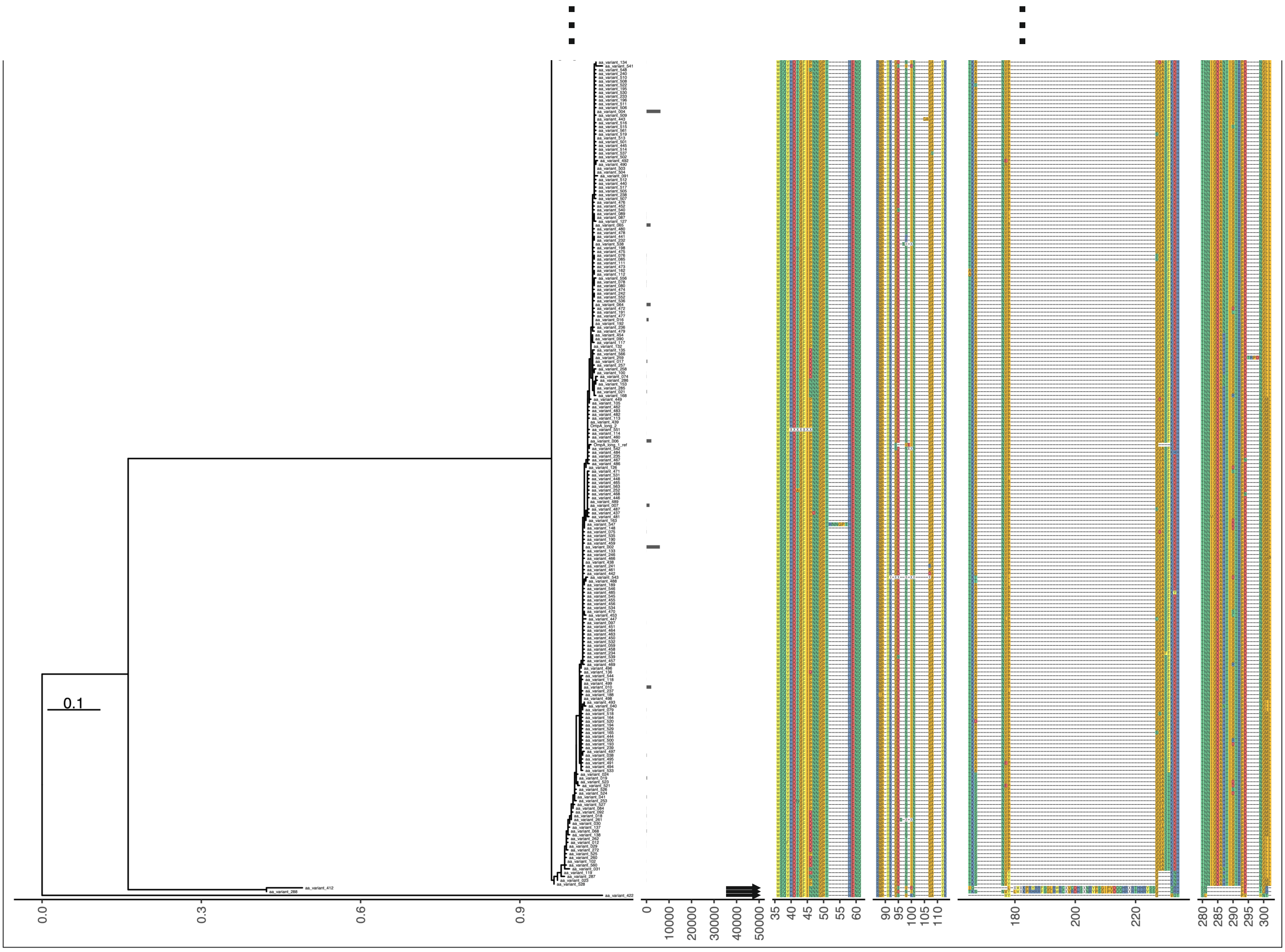

Supplementary Figure 1 | Sequence analysis of the OmpA protein variants in the 661k database. The left panel shows a phylogenetic tree of the unique 550 OmpA protein sequences, followed to the right by a bar plot depicting the number of isolates this protein variant was found in. The four panels on the right each depict the four extracellular loop regions of the underlying protein alignment. The major two groups in the tree correspond to the short (top, **a**) and long (bottom, **b**) OmpA isoforms. Additionally, four outliers (highlighted with arrows) can be identified: three variants at the base of the tree are very divergent as well as one variant in the short isoform-group which lacks sequence data for the loop regions. These four outliers have therefore been excluded from downstream analyses of the loop regions. For illustration purposes, the phylogenetic tree depicting OmpA-short and -long isoforms was divided onto two pages (**a** and **b**), with (**a**) showing OmpA-short and (**b**) OmpA-long isoforms, respectively.

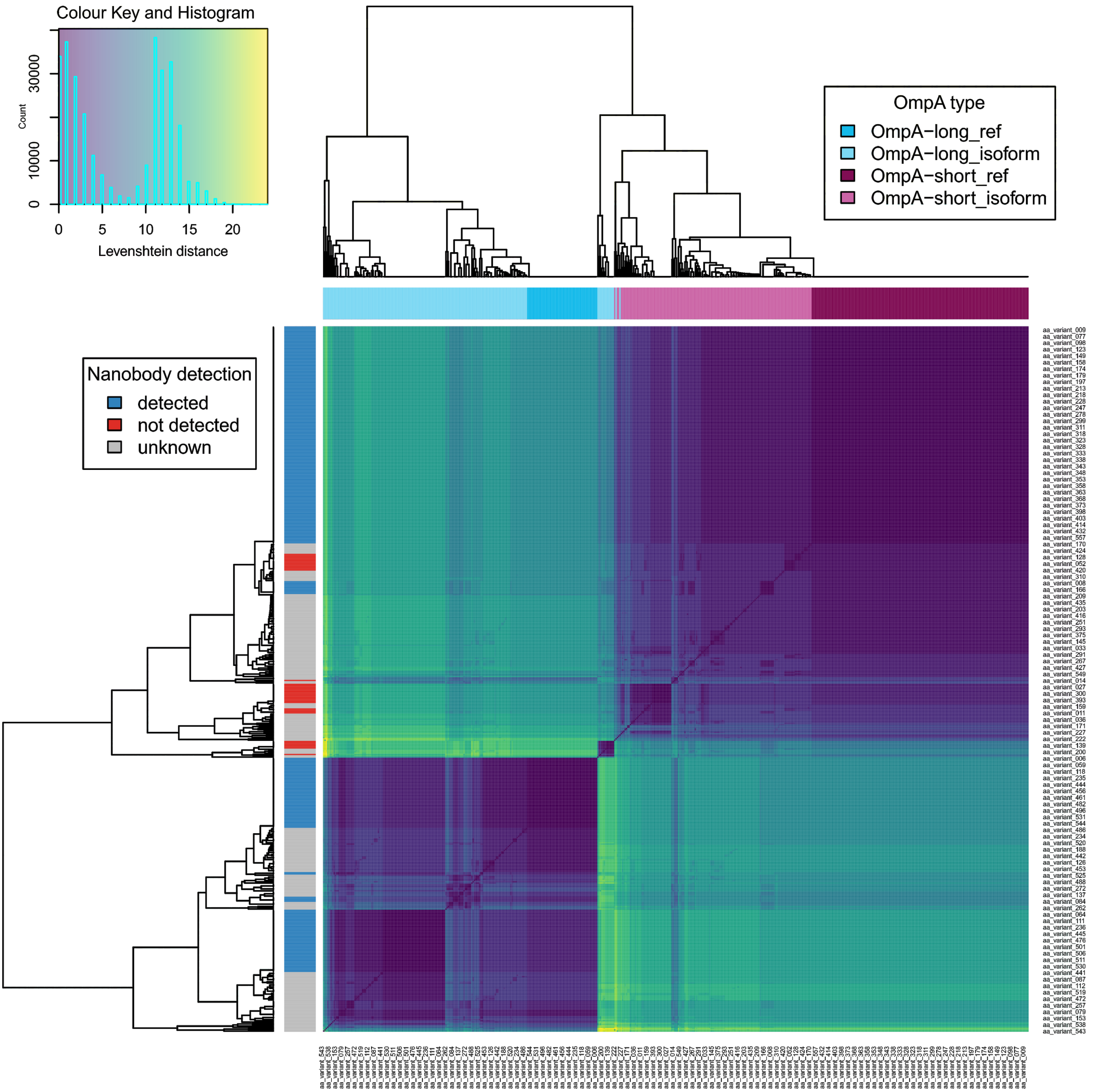

Supplementary Figure 2 |Hierarchical clustering of the concatenated extracellular loop alignments as shown in Supplementary Figure 1. On the x-axis, OmpA-long isoforms are colored in blue and OmpA-short isoforms in purple. The most frequent OmpA-long variant (OmpA-long_ref) and OmpA-short variant (OmpA-short_ref) are marked separately in darker color shades. On the y-axis, the results of the binding assay with Nb01 or Nb39 are marked according to the categories detected, not detected and unknown.

```
                                                  Loop 1
MC1061 wt      MKKTAIAIAVALAGFATVAQAAPKDNTWYTGAKLGWSQYHDTGFINNNGPTHENQLGAGAFGGYQVNPYVGFEMG 75
CS#1           MKKTAIAIAVALAGFATVAQAAPKDNTWYTGAKLGWSQYHDTGFINNNGPTHENQLGAGAFGGYQVNPYVGFEMG 75
CS#2           MKKTAIAIAVALAGFATVAQAAPKDNTWYTGAKLGWSQYHDTGFINNNGPTHENQLGAGAFGGYQVNPYVGFEMG 75
CS#3           MKKTAIAIAVALAGFATVAQAAPKDNTWYTGAKLGWSQYHDTGFINNNGPTHENQLGAGAFGGYQVNPYVGFEMG 75
CS#4           MKKTAIAIAVALAGFATVAQAAPKDNTWYTGAKLGWSQYHDTGFIDNNGPTHENQLGAGAFGGYQVNPYVGFEMG 75
               *********************************************:*****************************

                     Loop 2                                         Loop 3
MC1061 wt      YDWLGRMPYKGSVENGAYKAQGVQLTAKLGYPITDDLDIYTRLGGMVWRADTKSNVYGKNHDTGVSPVFAGGVEY 150
CS#1           YDWLGRMPYKGSVENGAYKAQGVQLTAKLGYPITDDLDVYTRLGGMVWRADTKSNVYGKNHDTGVSPVFAGGVEY 150
CS#2           YDWLGRMPYKGSVENGAYKAQGVQLTAKLGYPITDDLDIYTRLGGMVWRADTKSNVYGKNHDTGVSPVFAGGVEY 150
CS#3           YDWLGRMPYKGSVENGAYKAQGVQLTAKLGYPITDDLDVYTRLGGMVWRADTKSNVYGKNHDTGVSPVFAGGVEY 150
CS#4           YDWLGRMPYKGSVENGAYKAQGVQLTAKLGYPITDDLDVYTRLGGMVWRADTKSNVYGKNHDTGVSPVFAGGVEY 150
               **************************************:************************************

                              Loop 4                 Periplasmic domain
MC1061 wt      AITPEIATRLEYQWTNNIGDAHTIGTRPDNGMLSLGVSYRFGQGEAAPVVAPAPAPAPEVQTKHFTLKSDVLFNF 225
CS#1           AITPEIATRLEYQWTNNIGDAHTIGTRPDNGMLSLGVSYRFGQGEAAPVVAPAPAPAPEVQTKHFTLKSDVLFTF 225
CS#2           AITPEIATRLEYQWTNNIGDAHTIGTRPDNGMLSLGVSYRFGQGEAAPVVAPAPAPAPEVQTKHFTLKSDVLFNF 225
CS#3           AITPEIATRLEYQWTNNIGDAHTIGTRPDNGMLSLGVSYRFGQGEAAPVVAPAPAPAPEVQTKHFTLKSDVLFTF 225
CS#4           AITPEIATRLEYQWTNNIGDAHTIGTRPDNGMLSLGVSYRFGQGEVAPVVAPAPAPAPEVQTKHFTLKSDVLFTF 225
               *********************************************.***************************.*

MC1061 wt      NKATLKPEGQAALDQLYSQLSNLDPKDGSVVVLGYTDRIGSDAYNQGLSERRAQSVVDYLISKGIPADKISARGM 300
CS#1           NKATLKPEGQAALDQLYSQLSNLDPKDGSVVVLGYTDRIGSDAYNQGLSERRAQSVVDYLISKGIPADKISARGM 300
CS#2           NKATLKPEGQAALDQLYSQLSNLDPKDGSVVVLGYTDRIGSDAYNQGLSERRAQSVVDYLISKGIPADKISARGM 300
CS#3           NKATLKPEGQAALDQLYSQLSNLDPKDGSVVVLGYTDRIGSDAYNQALSERRAQSVVDYLISKGIPADKISARGM 300
CS#4           NKATLKPEGQAALDQLYSQLSNLDPKDGSVVVLGYTDRIGSDAYNQALSERRAQSVVDYLISKGIPADKISARGM 300
               **********************************************.****************************

MC1061 wt      GESNPVTGNTCDNVKQRAALIDCLAPDRRVEIEVKGIKDVVTQPQA 346
CS#1           GESNPVTGNTCDNVKQRAALIDCLAPDRRVEIEVKGIKDVVTQPQA 346
CS#2           GESNPVTGNTCDNVKQRAALIDCLAPDRRVEIEVKGIKDVVTQPQA 346
CS#3           GESNPVTGNTCDNVKQRAALIDCLAPDRRVEIEVKGIKDVVTQPQA 346
CS#4           GESNPVTGNTCDNVKQRAALIDCLAPDRRVEIEVKGIKDVVTQPQA 346
               **********************************************
```

Supplementary Figure 3 | Alignment of OmpA-short sequences of *E. coli* strains used in this study. The reference OmpA-short sequence present in *E. coli* MC1061 wt (bold) was used for alpaca immunizations and nanobody selections. The four external loops and the start of the periplasmic domain are marked. OmpA-short residues of clinical strains that differ from the reference sequence are highlighted in bold.

```
                                                   Loop 1
CS#11    MKKTAIAIAVALAGFATVAQAAPKDNTWYTGAKLGWSQYHDTGFIPNNGPTHENQLGAGAFGGYQVNPYVGFEMG 75
CS#5     MKKTAIAIAVALAGFATVAQAAPKDNTWYTGAKLGWSQYHDTGFIPNNGPTHENQLGAGAFGGYQVNPYVGFEMG 75
CS#6     MKKTAIAIAVALAGFATVAQAAPKDNTWYTGAKLGWSQYHDTGFIPNNGPTHENQLGAGAFGGYQVNPYVGFEMG 75
CS#7     MKKTAIAIAVALAGFATVAQAAPKDNTWYTGAKLGWSQYHDTGFIPNNGPTHENQLGAGAFGGYQVNPYVGFEMG 75
CS#8     MKKTAIAIAVALAGFATVAQAAPKDNTWYTGAKLGWSQYHDTGFIPNNGPTHENQLGAGAFGGYQVNPYVGFEMG 75
CS#9     MKKTAIAIAVALAGFATVAQAAPKDNTWYTGAKLGWSQYHDTGFIPNNGPTHENQLGAGAFGGYQVNPYVGFEMG 75
CS#10    MKKTAIAIAVALAGFATVAQAAPKDNTWYTGAKLGWSQYHDTGFIPNNGPTHENQLGAGAFGGYQVNPYVGFEMG 75
         ***************************************************************************

               Loop 2                                             Loop 3
CS#11    YDWLGRMPYKGDNINGAYKAQGVQLTAKLGYPITDDLDIYTRLGGMVWRADTKANVPGGASFKDHDTGVSPVFAG 150
CS#5     YDWLGRMPYKGDNINGAYKAQGVQLTAKLGYPITDDLDVYTRLGGMVWRADTKANVPGGASFKDHDTGVSPVFAG 150
CS#6     YDWLGRMPYKGDNINGAYKAQGVQLTAKLGYPITDDLDIYTRLGGMVWRADTKANVPGGASFKDHDTGVSPVFAG 150
CS#7     YDWLGRMPYKGDNINGAYKAQGVQLTAKLGYPITDDLDVYTRLGGMVWRADTKANVPGGASFKDHDTGVSPVFAG 150
CS#8     YDWLGRMPYKGDNINGAYKAQGVQLTAKLGYPITDDLDIYTRLGGMVWRADTKANVPGGASFKDHDTGVSPVFAG 150
CS#9     YDWLGRMPYKGDNINGAYKAQGVQLTAKLGYPITDDLDVYTRLGGMVWRADTKANVPGGASFKDHDTGVSPVFAG 150
CS#10    YDWLGRMPYKGDNINGAYKAQGVQLTAKLGYPITDDLDVYTRLGGMVWRADTKANVPGGASFKDHDTGVSPVFAG 150
         ******************************************:************************************

                          Loop 4                Periplasmic domain
CS#11    GVEYAITPEIATRLEYQWTNNIGDAHTIGTRPDNGMLSLGVSYRFGQGEAAPVVAPAPAPAPEVQTKHFTLKSDV 225
CS#5     GVEYAITPEIATRLEYQWTNNIGDANTIGTRPDNGLLSLGVSYRFGQGEAAPVVAPAPAPAPEVQTKHFTLKSDV 225
CS#6     GVEYAITPEIATRLEYQWTNNIGDAHTIGTRPDNGMLSLGVSYRFGQGEVAPVVAPAPAPAPEVQTKHFTLKSDV 225
CS#7     GVEYAITPEIATRLEYQWTNNIGDAHTIGTRPDNSMLSLGVSYRFGQGEAAPVVAPAPAPAPEVQTKHFTLKSDV 225
CS#8     GVEYAITPEIATRLEYQWTNNIGDAHTIGTRPDNGMLSLGVSYRFGQGEAAPVVAPAPAPAPEVQTKHFTLKSDV 225
CS#9     GVEYAITPEIATRLEYQWTNNIGDANTIGTRPDNGLLSLGVSYRFGQGEAAPVVAPAPAPAPEVQTKHFTLKSDV 225
CS#10    GVEYAITPEIATRLEYQWTNNIGDAHTIGTRPDNGMLSLGVSYRFGQGEAAPVVAPAPAPAPEVQTKHFTLKSDV 225
         *************************:********.:*************.*************************

CS#11    LFTFNKATLKPEGQAALDQLYSQLSNLDPKDGSVVVLGYTDRIGSDAYNQALSERRAQSVVDYLISKGIPADKIS 300
CS#5     LFNFNKATLKPEGQAALDQLYSQLSNLDPKDGSVVVLGYTDRIGSDAYNQGLSERRAQSVVDYLISKGIPADKIS 300
CS#6     LFTFNKATLKPEGQAALDQLYSQLSNLDPKDGSVVVLGYTDRIGSDAYNQALSERRAQSVVDYLISKGIPADKIS 300
CS#7     LFNFNKATLKPEGQAALDQLYSQLSNLDPKDGSVVVLGYTDRIGSDAYNQGLSERRAQSVVDYLISKGIPADKIS 300
CS#8     LFTFNKATLKPEGQAALDQLYSQLSNLDPKDGSVVVLGYTDRIGSDAYNQALSERRAQSVVDYLISKGIPADKIS 300
CS#9     LFNFNKATLKPEGQAALDQLYSQLSNLDPKDGSVVVLGYTDRIGSDAYNQGLSERRAQSVVDYLISKGIPADKIS 300
CS#10    LFNFNKATLKPEGQAALDQLYSQLSNLDPKDGSVVVLGYTDRIGSDAYNQGLSERRAQSVVDYLISKGIPADKIS 300
         **.***********************************************.************************

CS#11    ARGMGESNPVTGNTCDNVKQRAALIDCLAPDRRVEIEVKGIKDVVTQPQA 350
CS#5     ARGMGESNPVTGNTCDNVKQRAALIDCLAPDRRVEIEVKGIKDVVTQPQA 350
CS#6     ARGMGESNPVTGNTCDNVKQRAALIDCLAPDRRVEIEVKGIKDVVTQPQA 350
CS#7     ARGMGESNPVTGNTCDNVKQRAALIDCLAPDRRVEIEVKGIKDVVTQPQA 350
CS#8     ARGMGESNPVTGNTCDNVKQRAALIDCLAPDRRVEIEVKGIKDVVTQPQA 350
CS#9     ARGMGESNPVTGNTCDNVKQRAALIDCLAPDRRVEIEVKGIKDVVTQPQA 350
CS#10    ARGMGESNPVTGNTCDNVKQRAALIDCLAPDRRVEIEVKGIKDVVTQPQA 350
         **************************************************
```

Supplementary Figure 4 | Alignment of OmpA-long sequences of *E. coli* strains used in this study. The reference OmpA-long sequence present in clinical strain #11 (CS#11) was used for alpaca immunizations and nanobody selections. The four external loops and the start of the periplasmic domain are marked. OmpA-long residues that differ from the reference sequence are highlighted in bold.

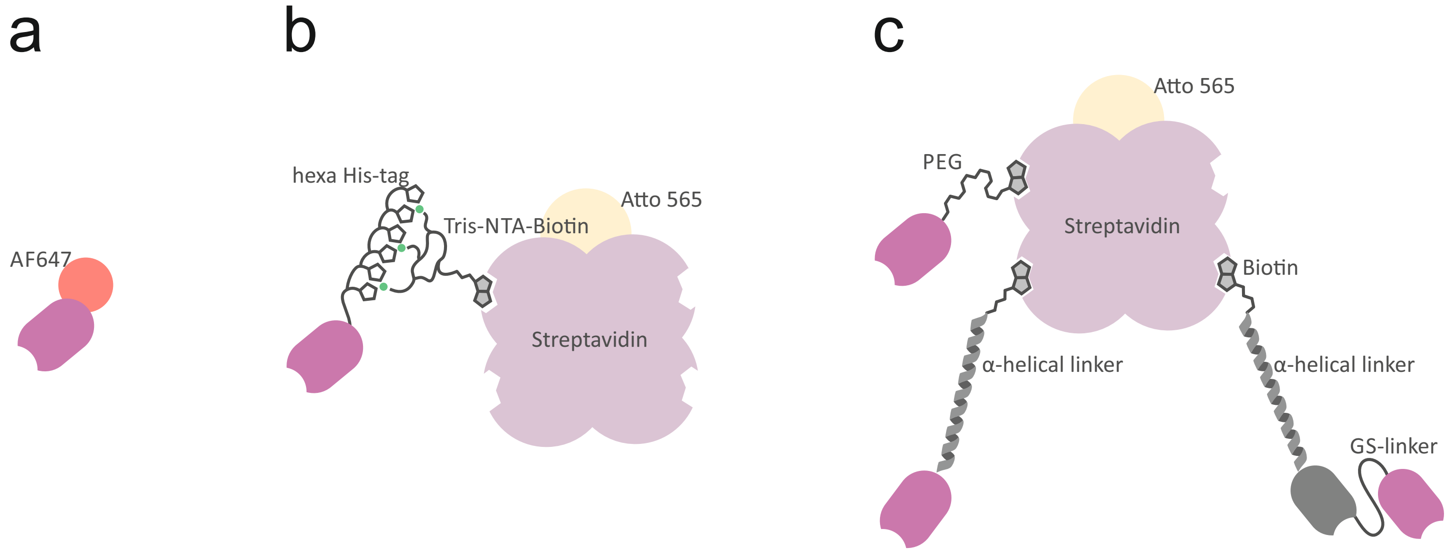

Supplementary Figure 5 | Assay formats for nanobody-mediated cellular staining. Nanobodies were labeled either directly (**a**) or indirectly (**b** and **c**). **a** A small fluorescent dye such as AF647, was conjugated to the nanobody by an additionally introduced C-terminal Cys residue via Cys-maleimide coupling. **b** Nanobodies were labeled via the C-terminal hexa-His-tag with Atto595-conjugated streptavidin. Ni^{2+}-ions coordinate both His residue and NTA of the Tris-NTA-Biotin molecule. **c** Nanobodies either expressed an C-terminal Avi-tag for *in vitro* biotinylation through BirA or a C-terminal Cys-residue that was used to couple maleimide-PEG_{11}-Biotin. The biotinylated nanobody was indirectly labeled using Atto595-conjugated streptavidin.

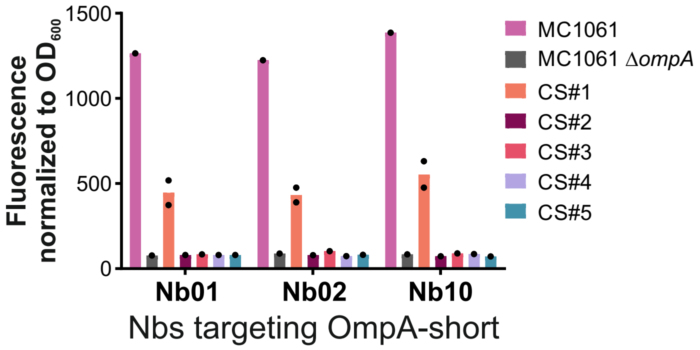

Supplementary Figure 6 | Fluorescent staining of *E. coli* strains using enlarged nanobodies. Three OmpA-short nanobodies were labeled via their C-terminal His-tags by a bulky NTA-Biotin-Streptavidin-Atto565 moiety (see also Supplementary Fig. 5). Cellular binding was assessed against lab strain *E. coli* MC1061 or the indicates clinical strains (CS). *E. coli* MC1061 lacking *ompA* served as negative control.

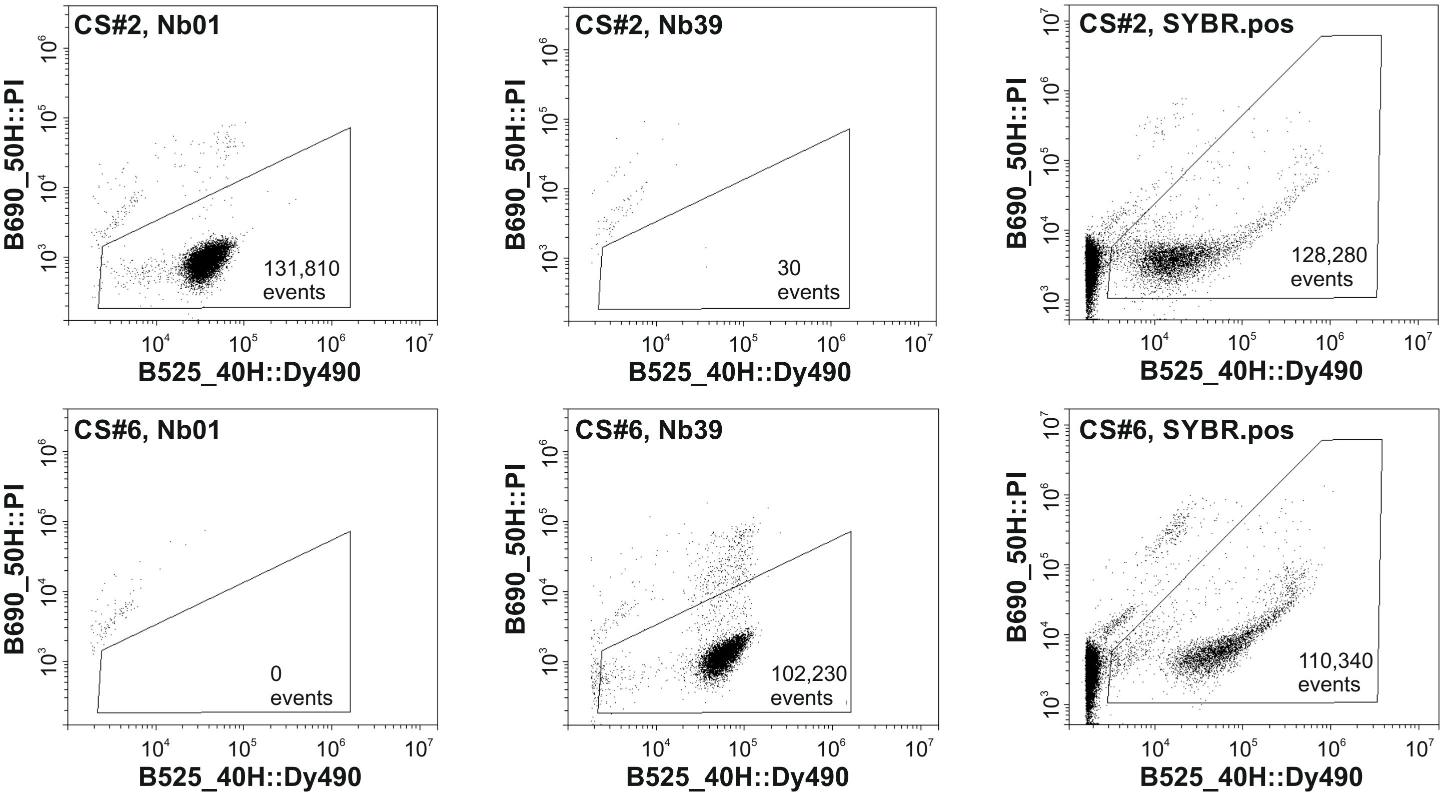

Supplementary Figure 7 | High throughput nanobody binding assay using flow cytometry. Exemplar dot-plots of flow cytometry analysis for *E. coli* CS#2 and CS#6. The number of events detected within the gate window are indicated. Nanobodies were labeled with Dy490. SYBR Green staining was used to count the total number of bacteria analyzed by flow cytometry. Data evaluation for all bacterial strains analyzed by this assay are shown in Supplementary Table 8.

```
MC1061 wt     -----WSQYHDTGFINNNGPTHENQ-----RMPYKGSVENGAYK-----TKSNVYGKNH-----TNNIGDAHTIGTRPDNGML
OmpA-ND#1     -----WSQYHDTGFINNNGPTHENQ-----RMPYKGSVENGAYK-----TKSNFDGKNH-----TNNIGDAHTIGTRPDNGML
OmpA-ND#5     -----WSQYHDTGFIDNNGPTHENQ-----RMPYKGSVENGAYK-----TKSNFDGKNH-----TNNIGDAHTIGTRPDNGML
OmpA-ND#3     -----WSQYHDTGFINNNGPTHENQ-----RMPYKGSVKNGAYK-----TKSNVYGKNH-----TNNIGDAHTIGTRPDNGML
OmpA-ND#4     -----WSQYHDTGFINNNGPTHENQ-----RMPYKGDNINGAYK-----TKSNVYGKNH-----TNNIGDAHTIGTRPDNGML
                   **********:*********     ******.  *****     ****. ****     *******************

CS#11         -----WSQYHDTGFIPNNGPTHENQ-----RMPYKGDNINGAYK-----TKAN-VPGGASFKDH-----TNNIGDAHTIGTRPDNGML
OmpA-ND#2     -----WSQYHDTGFIDNNGPTHENQ-----RMPYKGSVENGAYK-----TKAHNNVTGESEKNH-----TNNIGDAHTIGTRPDNGLL
                   ********** *********     ******.  *****     ***:    * * *:*     *****************:*
```

Supplementary Figure 8 | Sequence analysis of 5 most frequent OmpA variants not detected by Nb01 and Nb39. Experimentally determined binding of Nb01 and Nb39 to OmpA variants was matched with available OmpA loop variants in the 661k database and the Swiss database. Four of those belong to the OmpA-short variant, while one belongs to OmpA-long. Loop sequences of the most frequent undetected variants were aligned to the respective OmpA-short (top) or OmpA-long(bottom) reference (indicated in bold letters) using Clustal Omega. Residues that differ from the reference sequence are highlighted in bold.

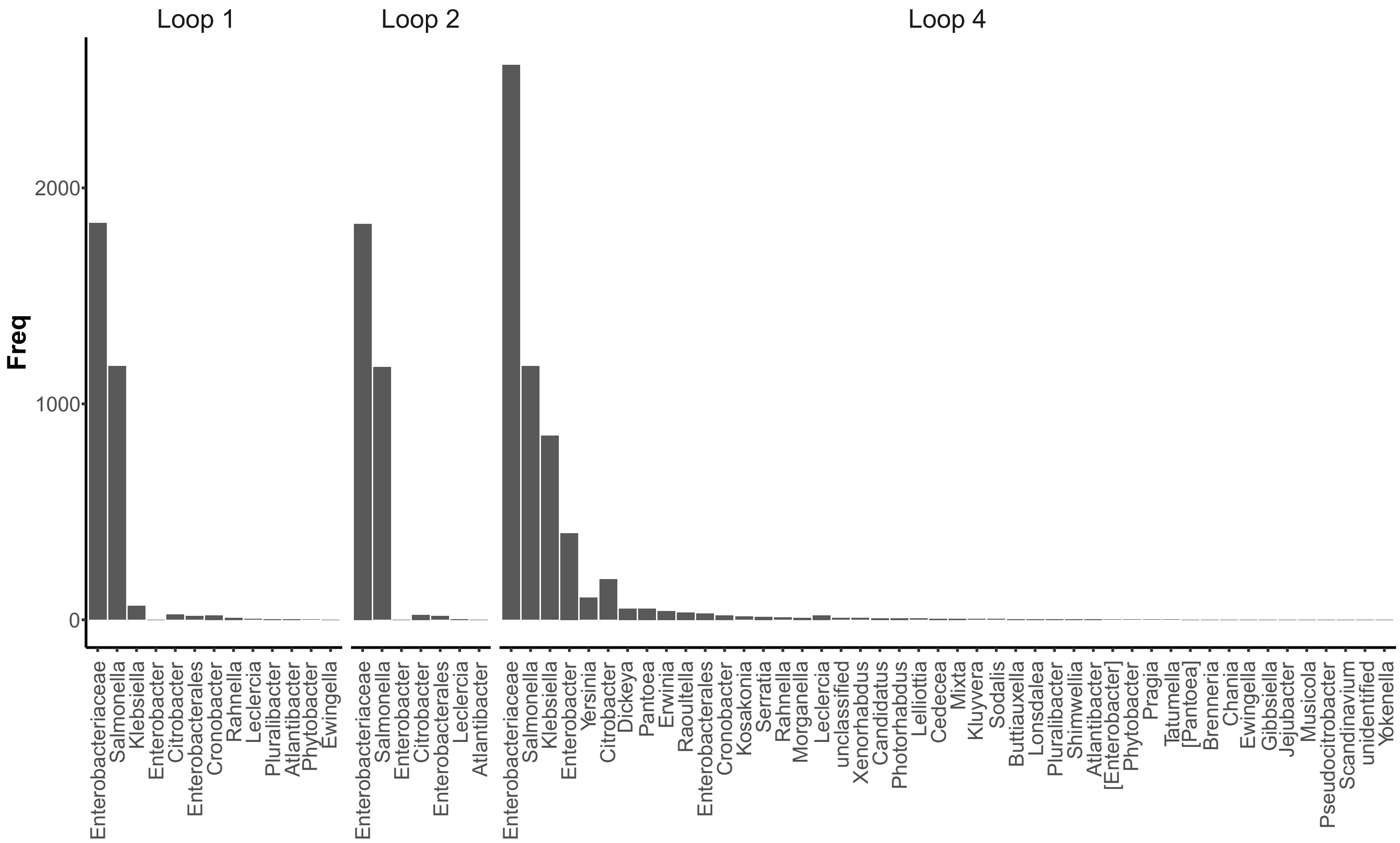

Supplementary Figure 9 | *In silico* specificity analysis of OmpA nanobodies. A sequence analysis using hidden Markov models (see methods) was performed to assess potential cross-reactions of our nanobodies by screening for protein sequences in RefSeq similar to each extracellular OmpA loop. The plot shows the number of proteins containing similar sequences to the extracellular loops of *E. coli* OmpA in other bacteria. The taxonomic grouping is based on the assignment of the corresponding RefSeq entry, simplified to the genus level in most cases. No matches were found for loop 3, all other matches were within OmpA homologues.

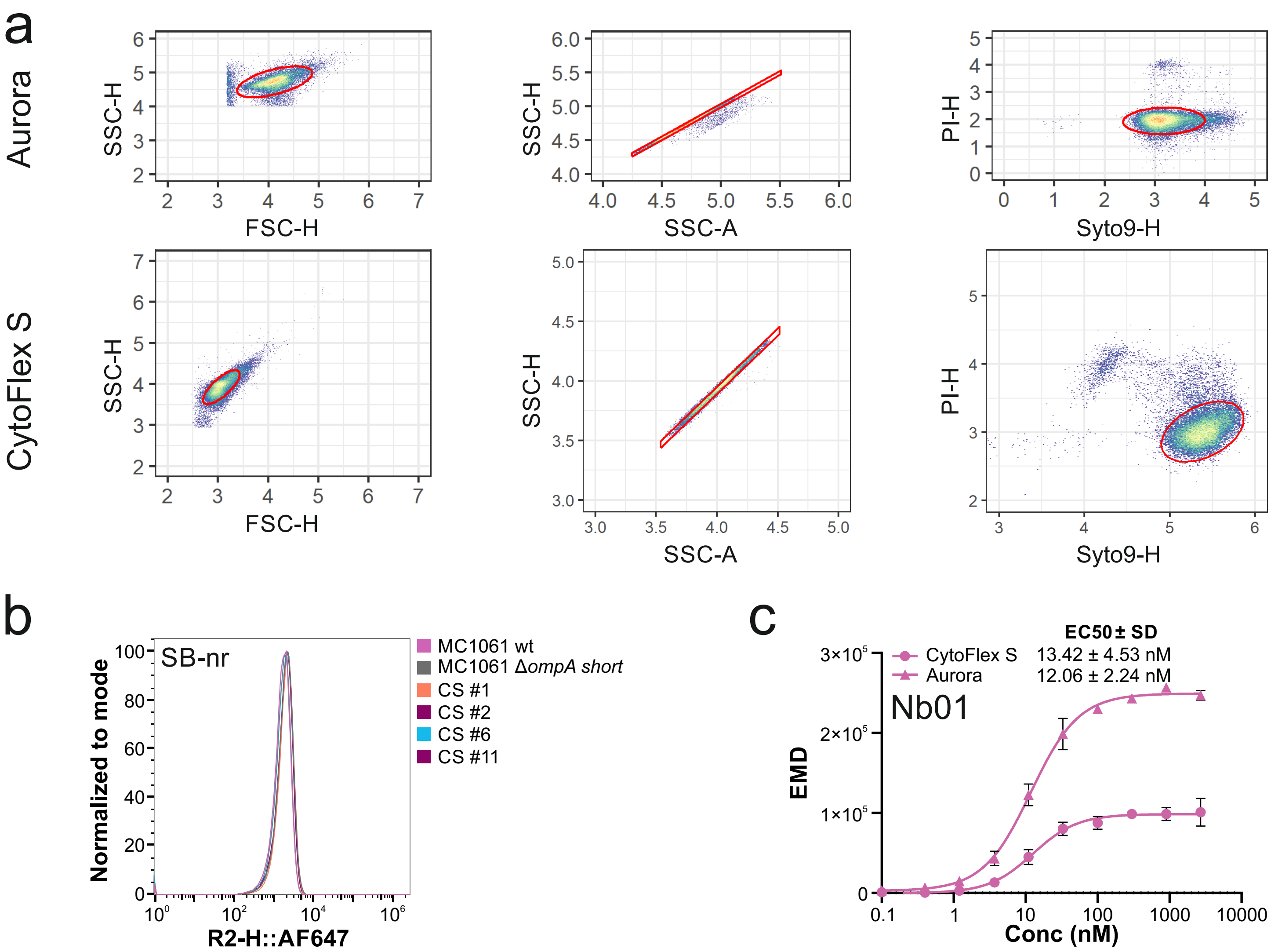

Supplementary Figure 10 | Characterization of Nb01 using flow cytometry. **a** Gating strategy in flow cytometry using the Aurora or the CytoFlex S instrument. **b** A non-randomized synthetic nanobody (Sb-nr) labeled with AF647 was used to assess cellular background binding for the calculation of earth mover's distance (EMD) values. Shown are flow cytometry histogram of the AF647 signal in the gated live population of the indicated bacterial strains. Measurements were performed on the Aurora flow cytometer using unfixed cells. **c** Determination of EC50 values for Nb01-AF647 binding to *E. coli* MC1061, measured on either a CytoFlex S or an Aurora flow cytometer, respectively. Data shown in (**c**) were fitted with the Hill equation to determine EC50 values. Representative data of three biological replicates are shown. Errors correspond to standard deviations of three biological replicates, which correspond to three independently inoculated cultures grown and processed in parallel.

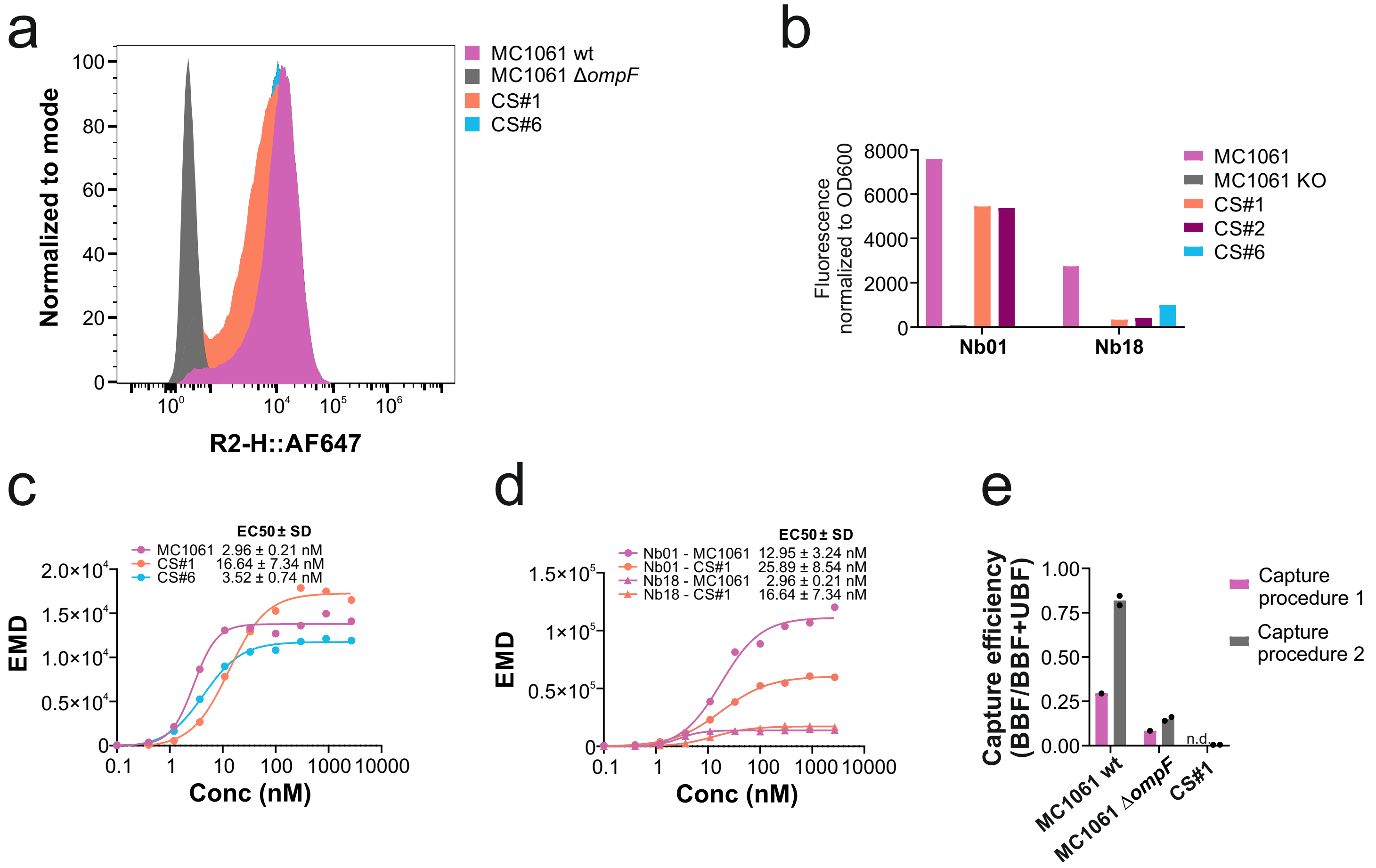

Supplementary Figure 11 | Characterization of Nb18 targeting OmpF. a Analysis of AF647-labeled Nb18 using flow cytometry. Nb18 binding to lab strain *E. coli* MC1061 was compared to clinical strains CS#1 and CS#2. *E. coli* MC1061 *ΔompF* served as negative control. **b** Comparative cellular staining of indicated *E. coli* strains using AF647-labeled Nb01 (against OmpA-short) or Nb18 (against OmpF). MC1061 KO denotes the lab strain being deleted for the targeted OMP. **c** and **d** Concentration-dependent binding of Nb18-AF647 against the indicated strains (**c**) and in comparison with Nb01-AF647 (**d**). Data were fitted with the Hill equation to determine EC50 values. Representative data of three biological replicates are shown. Errors correspond to standard deviations of three biological replicates, which correspond to three independently inoculated cultures grown and processed in parallel. **e** Capture of indicated strains using Nb18-PEG11-Biotin and following capture procedure 1 or 2 (see Fig. 6a). Capture was performed for 2 h.

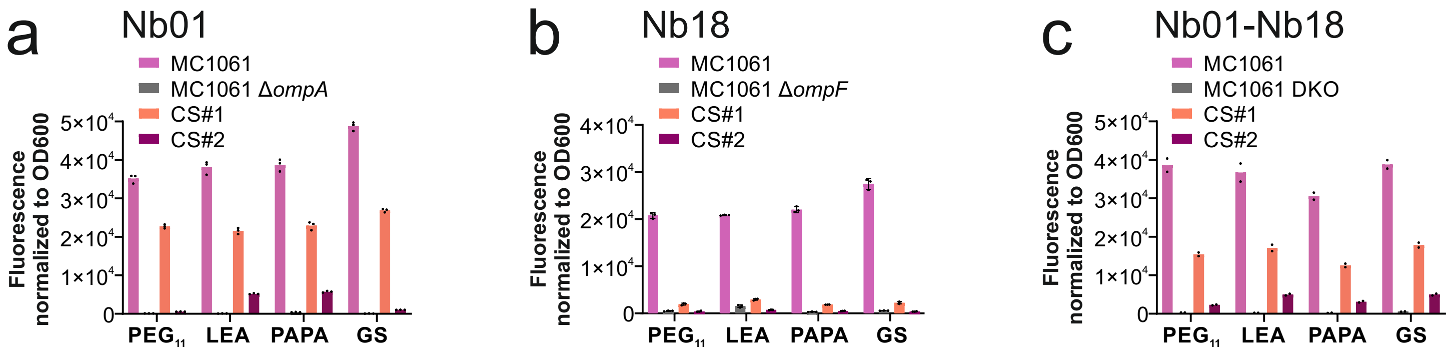

Supplementary Figure 12 | Testing nanobody constructs with different linker formats for fluorescent staining of E. coli strains. All constructs have one or two nanobodies at the N-terminus, followed by linkers of different lengths and flexibilities and bearing a biotin moiety placed most distantly from the N-terminal nanobody (see also Fig. 6b). Constructs were enlarged by forming a complex with bulky streptavidin-Atto565, allowing for fluorescent detection (see also Supplementary Fig. 5). *E. coli* cells were all grown in LB. **a** Cellular staining using Nb01 (targeting OmpA-short) modified with PEG11-Biotin (short linker), or extended by longer α-helical (PAPA, LEA) or flexible (GS) polypeptide linkers followed by a biotinylated Avi-tag. **b** Analogous to (**a**) but having Nb18 (targeting OmpF) at the N-terminus. **c** Analogous to (**a**) and (**b**), but having Nb01 fused to Nb18 with a flexible GS-linker at the N-terminus. Note that the Nb01-GS-Nb18-LEA-Biotin construct was used for the capture assay shown in Fig. 6f.

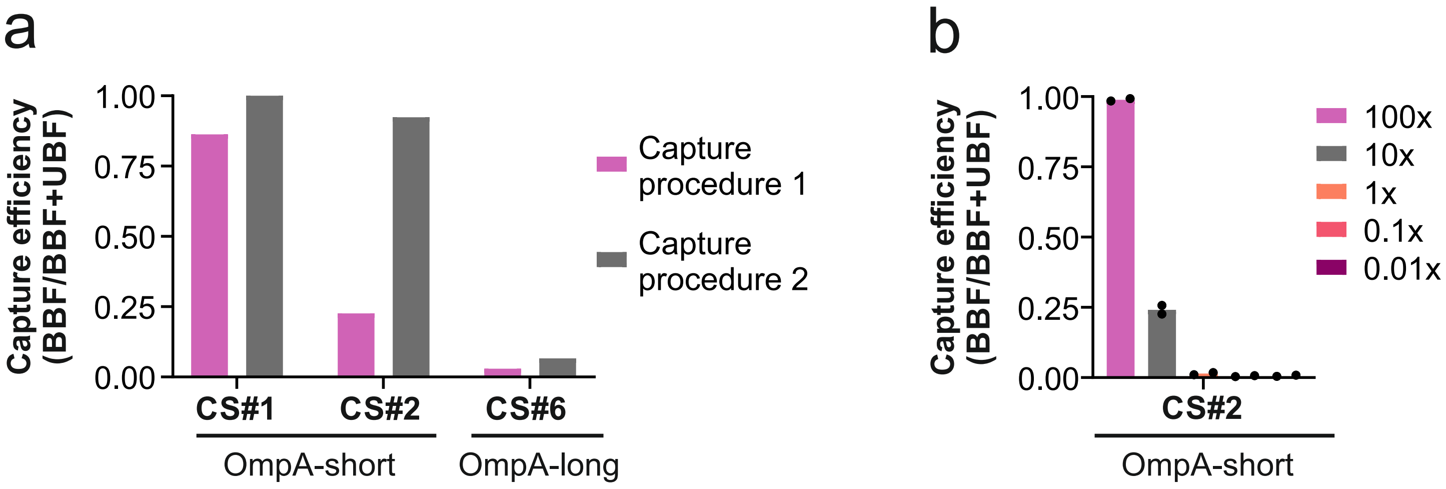

Supplementary Figure 13 | Capture of clinical *E. coli* strains using heavy-chain antibodies (hcAb). a Direct comparison of capture procedure 1 (bacterial capture with magnetic beads functionalized with hcAB) and capture procedure 2 (staining of cells with biotinylated hcAB, followed by capture with strepatavidin-coated magnetic beads) (see also Fig. 6a). Capture was performed with 4Nb01-hcAb (specific to OmpA-short) for 1 h with indicated clinical strains cultured in LB supplemented with 0.5 mM EDTA for CS#1 and CS#2 or 0.25 mM EDTA for CS#6. **b** Capture of CS#2 grown in LB supplemented with 0.5 mM EDTA and using 4Nb01-hcAb according to capture procedure 2. The estimated molar ratio of 4Nb01-hcAb in capture buffer versus OmpA-short present on the bacterial cell surface was varied from 100 fold to 0.01 fold by adding different amounts of 4Nb01-hcAb to a fixed amount of cells (for details, see methods).

PelB leader sequence, immunoglobulin secretion signal
Nb sequence
Linker ((GGGGS)$_4$, LEA, PAPA, hinge)
Avi-tag
3C recognition site
Myc-tag
His-tag

Nb01-Cys

ATGAGTAAATATCTGCTGCCGACCGCAGCAGCGGGTCTGCTGCTGCTGGCAGCCCAGCCTGCAATGGCCGGCTCT
TCAAGTCAAGTCCAGTTAGTCGAATCTGGAGGCGGGCTTGTTCAGCCCGGCGGCTCCCTGCGCCTGTCCTGCGTT
GTTAGCGGGACAGGGTTCACATTTAGTAAATCACCAATGAGCTGGGCGCGGCAGGCGCCAGGTAAGGAGCGTGAA
TGGGTTTCTGCGATATTCGCGGATTCAAGTACATACTACAGTGATAGCGTGCGGGGACGTTTTACTATTAGTCGT
GATAATGCGAAAAATACGGTATACCTTCAGATGAATAATGTTAAACCGGAAGATACCGCAGTATACTACTGCGGC
CATCGTAGATTGGGTAAAACTACGTACGATTATAGAGGAAAAGGTACGCGCGTAACCGTTAGCGCAGGAAGAGCT
TGTGGCGAACAAAAACTCATCTCAGAAGAGGATCTGAATAGCGCCGTCGACCATCATCATCATCATCATTGA

GSSSQVQLVESGGGLVQPGGSLRLSCVVSGTGFTFSKSPMSWARQAPGKEREWVSAIFADSSTYYSDSVRGRFTI
SRDNAKNTVYLQMNNVKPEDTAVYYCGHRRLGKTTYDYRGKGTRVTVSAGRACGEQKLISEEDLNSAVDHHHHHH
*

Nb18-Cys

ATGAGTAAATATCTGCTGCCGACCGCAGCAGCGGGTCTGCTGCTGCTGGCAGCCCAGCCTGCAATGGCCGGCTCT
TCAAGTCAGCGGCAGCTGGTAGAATCGGGTGGGGGTCTCGTGCAGGCCGGTGGCTCTTTAACGCTGAGTTGTAGT
GCTACTGGAACGGTGCCGCGGATTGATGCTATGGGGTGGTATCGTAGGAGCACAGGTAAAAAACGAGAACAGGTG
GCGTCTGTTGGCCGTGGCGGTAGGACGAATTATAGCGATTCTGCCAAAGGACGTTTTACAATCTCCCGCAACGGC
AACACGGTAACCCTGCAAATGACCTCTCTTAAACCGGAGGATACAGACGTGTACTTTTGCAACGCACTGAAATAT
GGACGGAATGCCGATTACGACGATTATTGGGGTCGCGGTACTCGGGTGACAGTGTCGGCAGGAAGAGCTTGTGGC
GAACAAAAACTCATCTCAGAAGAGGATCTGAATAGCGCCGTCGACCATCATCATCATCATCATTGA

GSSSQRQLVESGGGLVQAGGSLTLSCSATGTVPRIDAMGWYRRSTGKKREQVASVGRGGRTNYSDSAKGRFTISR
NGNTVTLQMTSLKPEDTDVYFCNALKYGRNADYDDYWGRGTRVTVSAGRACGEQKLISEEDLNSAVDHHHHHH*

Nb39-Cys

ATGAGTAAATATCTGCTGCCGACCGCAGCAGCGGGTCTGCTGCTGCTGGCAGCCCAGCCTGCAATGGCCGGCTCT
TCAAGTCAGCGTCAGCTGGTTGAAAGCGGTGGTGGTCTGGTTCATACCGGTGGTAGCCTGAAACTGAGCTGTGTT
CCGAATGGTAGCATTTTTAATTTTAATCCGATGGGTTGGTATCGTCAGGTTAGCGGTCAGCAGCGTGAACTGGTT
GCAACCCTGACCCGTGATGGTGTTGAAAATTATGCAAGCAGCGTTAAAGGTCGTTTTACCATTAGCCGTGATAGC
GCAAAAAATACCCTGTATCTGCAGATGACCGATGTTAAACCGGGTGATGCAGCAGTTTATATTTGTCATGCAAAT
TATCGTATTGGTCGTAATGATCTGCCGGTTTGGGGTAAAGGTACCCCGGTGACCGTGAGCGCAGGAAGAGCTTGT
GGCGAACAAAAACTCATCTCAGAAGAGGATCTGAATAGCGCCGTCGACCATCATCATCATCATCATTGA

GSSSQRQLVESGGGLVHTGGSLKLSCVPNGSIFNFNPMGWYRQVSGQQRELVATLTRDGVENYASSVKGRFTISR
DSAKNTLYLQMTDVKPGDAAVYICHANYRIGRNDLPVWGKGTPVTVSAGRACGEQKLISEEDLNSAVDHHHHHH*

Nb01-LEA-Avi-3C-His10

ATGAGTAAATATCTGCTGCCGACCGCAGCAGCGGGTCTGCTGCTGCTGGCAGCCCAGCCTGCAATGGCCGGCTCT
TCAAGTCAAGTCCAGTTAGTCGAATCTGGAGGCGGGCTTGTTCAGCCCGGCGGCTCCCTGCGCCTGTCCTGCGTT
GTTAGCGGGACAGGGTTCACATTTAGTAAATCACCAATGAGCTGGGCGCGGCAGGCGCCAGGTAAGGAGCGTGAA
TGGGTTTCTGCGATATTCGCGGATTCAAGTACATACTACAGTGATAGCGTGCGGGGACGTTTTACTATTAGTCGT
GATAATGCGAAAAATACGGTATACCTTCAGATGAATAATGTTAAACCGGAAGATACCGCAGTATACTACTGCGGC
CATCGTAGATTGGGTAAAACTACGTACGATTATAGAGGAAAAGGTACGCGCGTAACCGTTAGCGCAGGAAGAGCT
GCGGAAGCGGCCGCGAAAGAAGCGGCCGCGAAAGAAGCGGCCGCGAAAGAAGCGGCCGCGAAAGCGCTGGAAGCG
GAAGCGGCCGCGAAAGAAGCGGCCGCGAAAGAAGCGGCCGCGAAAGAAGCGGCCGCGAAAGCGGGTCTGAACGAT
ATCTTCGAAGCGCAGAAAATCGAATGGCACGAATTAGAAGTTTTGTTTCAAGGTCCACATCACCATCACCATCAT
CATCATCATCATTGA

GSSSQVQLVESGGGLVQPGGSLRLSCVVSGTGFTFSKSPMSWARQAPGKEREWVSAIFADSSTYYSDSVRGRFTI
SRDNAKNTVYLQMNNVKPEDTAVYYCGHRRLGKTTYDYRGKGTRVTVSAGRAAEAAAKEAAAKEAAAKEAAAKAL
EAEAAAKEAAAKEAAAKEAAAKAGLNDIFEAQKIEWHELEVLFQGPHHHHHHHHHH*

Nb01-PAPA-Avi-3C-His10

ATGAGTAAATATCTGCTGCCGACCGCAGCAGCGGGTCTGCTGCTGCTGGCAGCCCAGCCTGCAATGGCCGGCTCT
TCAAGTCAAGTCCAGTTAGTCGAATCTGGAGGCGGGCTTGTTCAGCCCGGCGGCTCCCTGCGCCTGTCCTGCGTT
GTTAGCGGGACAGGGTTCACATTTAGTAAATCACCAATGAGCTGGGCGCGGCAGGCGCCAGGTAAGGAGCGTGAA
TGGGTTTCTGCGATATTCGCGGATTCAAGTACATACTACAGTGATAGCGTGCGGGGACGTTTTACTATTAGTCGT
GATAATGCGAAAAATACGGTATACCTTCAGATGAATAATGTTAAACCGGAAGATACCGCAGTATACTACTGCGGC
CATCGTAGATTGGGTAAAACTACGTACGATTATAGAGGAAAAGGTACGCGCGTAACCGTTAGCGCAGGAAGAGCT
GGCGCGGCACCGGCTGCGGCACCGGCGAAACAGGAAGCGGCCGCGCCGGCACCGGCTGCGAAAGCGGAAGCGCCG
GCAGCGGCTCCGGCGGCGAAAGCGGGTCTGAACGATATCTTCGAAGCGCAGAAAATCGAATGGCACGAATTAGAA
GTTTTGTTTCAAGGTCCACATCACCATCACCATCATCATCATCATCATTGA

GSSSQVQLVESGGGLVQPGGSLRLSCVVSGTGFTFSKSPMSWARQAPGKEREWVSAIFADSSTYYSDSVRGRFTI
SRDNAKNTVYLQMNNVKPEDTAVYYCGHRRLGKTTYDYRGKGTRVTVSAGRAGAAPAAAPAKQEAAAPAPAAKAE
APAAAPAAKAGLNDIFEAQKIEWHELEVLFQGPHHHHHHHHHH*

Nb01-GS-Avi-3C-His10

ATGAGTAAATATCTGCTGCCGACCGCAGCAGCGGGTCTGCTGCTGCTGGCAGCCCAGCCTGCAATGGCCGGCTCT
TCAAGTCAAGTCCAGTTAGTCGAATCTGGAGGCGGGCTTGTTCAGCCCGGCGGCTCCCTGCGCCTGTCCTGCGTT
GTTAGCGGGACAGGGTTCACATTTAGTAAATCACCAATGAGCTGGGCGCGGCAGGCGCCAGGTAAGGAGCGTGAA
TGGGTTTCTGCGATATTCGCGGATTCAAGTACATACTACAGTGATAGCGTGCGGGGACGTTTTACTATTAGTCGT
GATAATGCGAAAAATACGGTATACCTTCAGATGAATAATGTTAAACCGGAAGATACCGCAGTATACTACTGCGGC
CATCGTAGATTGGGTAAAACTACGTACGATTATAGAGGAAAAGGTACGCGCGTAACCGTTAGCGCAGGAAGAGCT
GGCGGTGGCGGTAGCGGCGGTGGCGGTAGCGGCGGTGGCGGTAGCGGCGGTGGCGGTAGCGGTCTGAACGATATC
TTCGAAGCGCAGAAAATCGAATGGCACGAATTAGAAGTTTTGTTTCAAGGTCCACATCACCATCACCATCATCAT
CATCATCATTGA

GSSSQVQLVESGGGLVQPGGSLRLSCVVSGTGFTFSKSPMSWARQAPGKEREWVSAIFADSSTYYSDSVRGRFTI
SRDNAKNTVYLQMNNVKPEDTAVYYCGHRRLGKTTYDYRGKGTRVTVSAGRAGGGGSGGGGSGGGGSGGGGSGLN
DIFEAQKIEWHELEVLFQGPHHHHHHHHHH*

Nb01-(GGGGS)4-Nb18-Cys

ATGAGTAAATATCTGCTGCCGACCGCAGCAGCGGGTCTGCTGCTGCTGGCAGCCCAGCCTGCAATGGCCGGCTCT
TCAAGTCAAGTCCAGTTAGTCGAATCTGGAGGCGGGCTTGTTCAGCCCGGCGGCTCCCTGCGCCTGTCCTGCGTT
GTTAGCGGGACAGGGTTCACATTTAGTAAATCACCAATGAGCTGGGCGCGGCAGGCGCCAGGTAAGGAGCGTGAA
TGGGTTTCTGCGATATTCGCGGATTCAAGTACATACTACAGTGATAGCGTGCGGGGACGTTTTACTATTAGTCGT
GATAATGCGAAAAATACGGTATACCTTCAGATGAATAATGTTAAACCGGAAGATACCGCAGTATACTACTGCGGC
CATCGTAGATTGGGTAAAACTACGTACGATTATAGAGGAAAAGGTACGCGCGTAACCGTTAGCGCAGGTGGTGGC
GGTAGCGGCGGTGGCGGTTCTGGTGGTGGCGGTAGCGGCGGTGGCGGTAGTCAGCGGCAGCTGGTAGAATCGGGT
GGGGGTCTCGTGCAGGCCGGTGGCTCTTTAACGCTGAGTTGTAGTGCTACTGGAACGGTGCCGCGGATTGATGCT
ATGGGGTGGTATCGTAGGAGCACAGGTAAAAAACGAGAACAGGTGGCGTCTGTTGGCCGTGGCGGTAGGACGAAT
TATAGCGATTCTGCCAAAGGACGTTTTACAATCTCCCGCAACGGCAACACGGTAACCCTGCAAATGACCTCTCTT
AAACCGGAGGATACAGACGTGTACTTTTGCAACGCACTGAAATATGGACGGAATGCCGATTACGACGATTATTGG
GGTCGCGGTACTCGGGTGACAGTGTCGGCAGGAAGAGCTTGTGGCGAACAAAAACTCATCTCAGAAGAGGATCTG
AATAGCGCCGTCGACCATCATCATCATCATCATTGA

GSSSQVQLVESGGGLVQPGGSLRLSCVVSGTGFTFSKSPMSWARQAPGKEREWVSAIFADSSTYYSDSVRGRFTI
SRDNAKNTVYLQMNNVKPEDTAVYYCGHRRLGKTTYDYRGKGTRVTVSAGGGGSGGGGSGGGGSGGGGSQRQLVE
SGGGLVQAGGSLTLSCSATGTVPRIDAMGWYRRSTGKKREQVASVGRGGRTNYSDSAKGRFTISRNGNTVTLQMT
SLKPEDTDVYFCNALKYGRNADYDDYWGRGTRVTVSAGRACGEQKLISEEDLNSAVDHHHHHH*

Nb01-(GGGGS)4-Nb18-LEA-Avi-3C-His10

ATGAGTAAATATCTGCTGCCGACCGCAGCAGCGGGTCTGCTGCTGCTGGCAGCCCAGCCTGCAATGGCCGGCTCT
TCAAGTCAAGTCCAGTTAGTCGAATCTGGAGGCGGGCTTGTTCAGCCCGGCGGCTCCCTGCGCCTGTCCTGCGTT
GTTAGCGGGACAGGGTTCACATTTAGTAAATCACCAATGAGCTGGGCGCGGCAGGCGCCAGGTAAGGAGCGTGAA
TGGGTTTCTGCGATATTCGCGGATTCAAGTACATACTACAGTGATAGCGTGCGGGGACGTTTTACTATTAGTCGT
GATAATGCGAAAAATACGGTATACCTTCAGATGAATAATGTTAAACCGGAAGATACCGCAGTATACTACTGCGGC
CATCGTAGATTGGGTAAAACTACGTACGATTATAGAGGAAAAGGTACGCGCGTAACCGTTAGCGCAGGTGGTGGC
GGTAGCGGCGGTGGCGGTTCTGGTGGTGGCGGTAGCGGCGGTGGCGGTAGTCAGCGGCAGCTGGTAGAATCGGGT
GGGGGTCTCGTGCAGGCCGGTGGCTCTTTAACGCTGAGTTGTAGTGCTACTGGAACGGTGCCGCGGATTGATGCT
ATGGGGTGGTATCGTAGGAGCACAGGTAAAAAACGAGAACAGGTGGCGTCTGTTGGCCGTGGCGGTAGGACGAAT
TATAGCGATTCTGCCAAAGGACGTTTTACAATCTCCCGCAACGGCAACACGGTAACCCTGCAAATGACCTCTCTT
AAACCGGAGGATACAGACGTGTACTTTTGCAACGCACTGAAATATGGACGGAATGCCGATTACGACGATTATTGG
GGTCGCGGTACTCGGGTGACAGTGTCGGCAGGAAGAGCTGCGGAAGCGGCCGCGAAAGAAGCGGCCGCGAAAGAA
GCGGCCGCGAAAGAAGCGGCCGCGAAAGCGCTGGAAGCGGAAGCGGCCGCGAAAGAAGCGGCCGCGAAAGAAGCG
GCCGCGAAAGAAGCGGCCGCGAAAGCGGGTCTGAACGATATCTTCGAAGCGCAGAAAATCGAATGGCACGAATTA
GAAGTTTTGTTTCAAGGTCCACATCACCATCACCATCATCATCATCATCATTGA

GSSSQVQLVESGGGLVQPGGSLRLSCVVSGTGFTFSKSPMSWARQAPGKEREWVSAIFADSSTYYSDSVRGRFTI
SRDNAKNTVYLQMNNVKPEDTAVYYCGHRRLGKTTYDYRGKGTRVTVSAGGGGSGGGGSGGGGSGGGGSQRQLVE
SGGGLVQAGGSLTLSCSATGTVPRIDAMGWYRRSTGKKREQVASVGRGGRTNYSDSAKGRFTISRNGNTVTLQMT
SLKPEDTDVYFCNALKYGRNADYDDYWGRGTRVTVSAGRAAEAAAKEAAAKEAAAKEAAAKALEAEAAAKEAAAK
EAAAKEAAAKAGLNDIFEAQKIEWHELEVLFQGPHHHHHHHHHH*

Nb01-(GGGGS)4-Nb18-PAPA-Avi-3C-His10

ATGAGTAAATATCTGCTGCCGACCGCAGCAGCGGGTCTGCTGCTGCTGGCAGCCCAGCCTGCAATGGCCGGCTCT
TCAAGTCAAGTCCAGTTAGTCGAATCTGGAGGCGGGCTTGTTCAGCCCGGCGGCTCCCTGCGCCTGTCCTGCGTT
GTTAGCGGGACAGGGTTCACATTTAGTAAATCACCAATGAGCTGGGCGCGGCAGGCGCCAGGTAAGGAGCGTGAA
TGGGTTTCTGCGATATTCGCGGATTCAAGTACATACTACAGTGATAGCGTGCGGGGACGTTTTACTATTAGTCGT
GATAATGCGAAAAATACGGTATACCTTCAGATGAATAATGTTAAACCGGAAGATACCGCAGTATACTACTGCGGC
CATCGTAGATTGGGTAAAACTACGTACGATTATAGAGGAAAAGGTACGCGCGTAACCGTTAGCGCAGGTGGTGGC
GGTAGCGGCGGTGGCGGTTCTGGTGGTGGCGGTAGCGGCGGTGGCGGTAGTCAGCGGCAGCTGGTAGAATCGGGT
GGGGGTCTCGTGCAGGCCGGTGGCTCTTTAACGCTGAGTTGTAGTGCTACTGGAACGGTGCCGCGGATTGATGCT
ATGGGGTGGTATCGTAGGAGCACAGGTAAAAAACGAGAACAGGTGGCGTCTGTTGGCCGTGGCGGTAGGACGAAT
TATAGCGATTCTGCCAAAGGACGTTTTACAATCTCCCGCAACGGCAACACGGTAACCCTGCAAATGACCTCTCTT
AAACCGGAGGATACAGACGTGTACTTTTGCAACGCACTGAAATATGGACGGAATGCCGATTACGACGATTATTGG
GGTCGCGGTACTCGGGTGACAGTGTCGGCAGGAAGAGCTGGCGCGGCACCGGCTGCGGCACCGGCGAAACAGGAA
GCGGCCGCGCCGGCACCGGCTGCGAAAGCGGAAGCGCCGGCAGCGGCTCCGGCGGCGAAAGCGGGTCTGAACGAT
ATCTTCGAAGCGCAGAAAATCGAATGGCACGAATTAGAAGTTTTGTTTCAAGGTCCACATCACCATCACCATCAT
CATCATCATCATTGA

GSSSQVQLVESGGGLVQPGGSLRLSCVVSGTGFTFSKSPMSWARQAPGKEREWVSAIFADSSTYYSDSVRGRFTI
SRDNAKNTVYLQMNNVKPEDTAVYYCGHRRLGKTTYDYRGKGTRVTVSAGGGGSGGGGSGGGGSGGGGSQRQLVE
SGGGLVQAGGSLTLSCSATGTVPRIDAMGWYRRSTGKKREQVASVGRGGRTNYSDSAKGRFTISRNGNTVTLQMT
SLKPEDTDVYFCNALKYGRNADYDDYWGRGTRVTVSAGRAGAAPAAAPAKQEAAAPAPAAKAEAPAAAPAAKAGL
NDIFEAQKIEWHELEVLFQGPHHHHHHHHHH*

Nb01-(GGGGS)4-Nb18-GS-Avi-3C-His10

ATGAGTAAATATCTGCTGCCGACCGCAGCAGCGGGTCTGCTGCTGCTGGCAGCCCAGCCTGCAATGGCCGGCTCT
TCAAGTCAAGTCCAGTTAGTCGAATCTGGAGGCGGGCTTGTTCAGCCCGGCGGCTCCCTGCGCCTGTCCTGCGTT
GTTAGCGGGACAGGGTTCACATTTAGTAAATCACCAATGAGCTGGGCGCGGCAGGCGCCAGGTAAGGAGCGTGAA
TGGGTTTCTGCGATATTCGCGGATTCAAGTACATACTACAGTGATAGCGTGCGGGGACGTTTTACTATTAGTCGT
GATAATGCGAAAAATACGGTATACCTTCAGATGAATAATGTTAAACCGGAAGATACCGCAGTATACTACTGCGGC
CATCGTAGATTGGGTAAAACTACGTACGATTATAGAGGAAAAGGTACGCGCGTAACCGTTAGCGCAGGTGGTGGC
GGTAGCGGCGGTGGCGGTTCTGGTGGTGGCGGTAGCGGCGGTGGCGGTAGTCAGCGGCAGCTGGTAGAATCGGGT
GGGGGTCTCGTGCAGGCCGGTGGCTCTTTAACGCTGAGTTGTAGTGCTACTGGAACGGTGCCGCGGATTGATGCT
ATGGGGTGGTATCGTAGGAGCACAGGTAAAAAACGAGAACAGGTGGCGTCTGTTGGCCGTGGCGGTAGGACGAAT
TATAGCGATTCTGCCAAAGGACGTTTTACAATCTCCCGCAACGGCAACACGGTAACCCTGCAAATGACCTCTCTT
AAACCGGAGGATACAGACGTGTACTTTTGCAACGCACTGAAATATGGACGGAATGCCGATTACGACGATTATTGG
GGTCGCGGTACTCGGGTGACAGTGTCGGCAGGAAGAGCTGGCGGTGGCGGTAGCGGCGGTGGCGGTAGCGGCGGT
GGCGGTAGCGGCGGTGGCGGTAGCGGTCTGAACGATATCTTCGAAGCGCAGAAAATCGAATGGCACGAATTAGAA
GTTTTGTTTCAAGGTCCACATCACCATCACCATCATCATCATCATCATTGA

GSSSQVQLVESGGGLVQPGGSLRLSCVVSGTGFTFSKSPMSWARQAPGKEREWVSAIFADSSTYYSDSVRGRFTI
SRDNAKNTVYLQMNNVKPEDTAVYYCGHRRLGKTTYDYRGKGTRVTVSAGGGGSGGGGSGGGGSGGGGSQRQLVE
SGGGLVQAGGSLTLSCSATGTVPRIDAMGWYRRSTGKKREQVASVGRGGRTNYSDSAKGRFTISRNGNTVTLQMT
SLKPEDTDVYFCNALKYGRNADYDDYWGRGTRVTVSAGRAGGGGSGGGGSGGGGSGGGGSGLNDIFEAQKIEWHE
LEVLFQGPHHHHHHHHHH*

6Nb01-hcAb-Avi (Secretion signal – Nb01 - LEA – Nb02 – PAPA - Nb01 – LEA – Nb10 – PAPA – Nb02 – LEA – Nb01 - hinge – hIgG1-Fc – Avi)

ATGGACTGGACCTGGCGGGTGTTTTGCCTGTTGGCAGTCGCCCCTGGGGCTCACTCTTCCCAGGTGCAACTGGTGGAGAGTGG
AGGAGGACTCGTGCAGCCAGGGGGTTCATTGAGGTTGTCATGTGTCGTGAGTGGTACTGGATTTACATTCTCCAAGTCCCCTA
TGTCCTGGGCACGACAAGCGCCTGGAAAGGAACGCGAATGGGTCTCAGCAATATTCGCTGACAGCTCAACCTATTATTCCGAC
AGTGTCCGGGGGCGGTTTACAATCTCCCGCGATAACGCTAAAAACACTGTCTATCTGCAAATGAATAATGTGAAGCCAGAGGA
TACGGCCGTGTACTACTGTGGCCACCGCCGTTTGGGCAAAACCACTTATGATTACCGGGGCAAGGGGACCAGGGTGACAGTGA
GCTCAGCTGAGGCCGCGGCTAAGGAGGCCGCCGCCAAAGAGGCGGCCGCTAAAGAAGCTGCTGCCAAAGCATTAGAGGCTGAA
GCCGCGGCCAAGGAAGCCGCCGCTAAGGAGGCGGCAGCCAAGGAAGCAGCTGCAAAGGCGAGTCAAATGCAATTCGTTGAGTC
TGGGGGCGGGCTGGTCCAGCCCGGAGGCAGTCTGCGGCTGAGCTGCGTTGTGTCCGGGACGGGCTTCACCTTTTCCAAGTCAC
CTATGTCTTGGGCCAGACAAGCCCCTGGGAAGGAAAGAGAGTGGGTGTCTGCAATTTTTGCCGACAGCAGCACCTATTATGCC
GATTCAGTCAAGGGGAGATTCACAATTTCTCGGGACAATGCTAAAAATACGGTCTACCTTCAGATGAATGATGTCCAACCAGA
AGACTCCGCTGTCTATTATTGCGGACACAGAAGGCTCGGGAAACGGACCTACGATTACCGTGGGCAGGGCACACCAGTGACCG
TGAGTAGCGGCGCAGCCCCCGCAGCTGCCCCCGCCAAGCAGGAGGCCGCAGCCCCCGCCCCTGCAGCTAAGGCCGAAGCACCC
GCAGCAGCCCCCGCTGCTAAGGCAAGCCAGGTCCAGCTCGTCGAGTCCGGTGGTGGTCTTGTGCAGCCAGGCGGCTCTCTTAG
ACTCTCCTGTGTTGTATCCGGCACTGGCTTTACCTTTTCCAAGTCGCCCATGAGCTGGGCAAGGCAGGCTCCCGGGAAGGAGC
GAGAGTGGGTGTCCGCTATCTTTGCCGACAGCTCTACGTACTACTCTGATTCAGTGCGGGGACGGTTTACCATCTCTAGGGAC
AACGCCAAGAACACTGTCTACCTGCAGATGAACAACGTCAAACCAGAGGACACTGCCGTGTATTATTGCGGACACCGGAGGTT
GGGTAAGACAACCTACGATTACCGCGGGAAGGGGACCCGAGTGACCGTATCATCCGCTGAGGCTGCCGCTAAAGAGGCTGCCG
CGAAAGAAGCAGCCGCTAAAGAGGCAGCCGCAAAGGCTCTAGAGGCAGAGGCCGCGGCCAAAGAGGCAGCCGCCAAAGAGGCC
GCAGCAAAGGAGGCTGCTGCTAAAGCGTCACAAGGGCAGCTTGTTGAGTCCGGTGGTGGGCTCGTCCAACCCGGGGGTAGTCT
TCGCCTGTCTTGCACCGCTAGTGGCTTCACGTTCAGTTCCTACGCTATGACGTGGCACCGTCAAGCCCCAGGTAAGGAGAGGG
AACTGGTCGCCCTCATCACAAACGATGCCCGCACACGGTATGGCGACTTCGTTAAAGGGAGATTTACCATCTCTCGAGACAAC
GCTAAGAACACAATCTATCTACAGATGAATACACTCGCTCCGGAAGACACTGCATTATACTACTGTGGTGTTAGCCTGCTGGG
ACGCAATTATGGTCAGCACTGGGGAAAAGGAACAAGGGTGACTGTTTCATCTGGAGCTGCCCCAGCCGCCGCACCGGCGAAGC
AGGAAGCCGCGGCTCCAGCACCTGCAGCGAAGGCTGAAGCTCCAGCCGCCGCCCCTGCTGCAAAGGCCAGTCAGATGCAGTTT
GTGGAAAGTGGCGGAGGACTGGTTCAGCCTGGCGGCTCCCTCAGACTGTCTTGTGTGGTTAGCGGGACCGGCTTCACTTTCTC
AAAAAGCCCAATGTCTTGGGCAAGGCAGGCACCGGGAAAGGAGCGCGAGTGGGTAAGTGCGATTTTCGCAGACTCAAGCACAT
ACTACGCCGATAGCGTGAAAGGACGCTTCACGATTTCTAGAGACAACGCAAAGAACACTGTATACTTGCAGATGAACGACGTG
CAACCAGAAGATAGCGCCGTTTATTATTGTGGCCATCGCAGATTGGGCAAAAGAACGTATGATTACAGAGGCCAGGGCACCCC
CGTTACTGTATCGAGCGCCGAAGCTGCCGCCAAGGAGGCCGCCGCCAAGGAGGCTGCTGCCAAAGAGGCAGCCGCCAAAGCCC
TTGAAGCCGAAGCTGCAGCCAAGGAGGCAGCCGCGAAGGAAGCCGCCGCCAAGGAGGCCGCTGCAAAAGCTTCTCAGGTCCAG
CTGGTAGAATCCGGCGGAGGTCTAGTCCAGCCCGGGGGAAGCCTACGCCTGTCTTGCGTGGTGAGCGGCACCGGATTCACATT
TAGTAAGAGCCCGATGTCTTGGGCACGCCAAGCGCCAGGGAAAGAGAGGGAATGGGTATCCGCTATTTTCGCAGATAGCTCAA
CCTACTATTCCGATTCGGTTAGGGGGCGATTCACTATCAGCCGAGATAATGCAAAAAATACTGTATACCTGCAGATGAATAAT
GTGAAGCCAGAGGACACCGCCGTATATTACTGCGGACATCGGCGGCTTGGCAAGACCACTTACGACTATAGAGGCAAAGGGAC
TAGGGTGACCGTGTCCTCAGATAAGACTCACACATGCCCTCCGTGCCCTGCACCTGAGCTGCTGGGCGGCCCCAGCGTGTTCC
TCTTCCCCCCTAAGCCAAAAGATACTTTAATGATATCTCGTACCCCCGAAGTGACCTGTGTTGTGGTGGATGTCTCTCATGAA
GACCCTGAGGTTAAGTTTAACTGGTACGTCGACGGTGTCGAGGTGCATAATGCTAAGACCAAACCCCGAGAGGAACAGTACAA
TAGCACATACAGAGTCGTCAGTGTGCTGACAGTGCTGCACCAGGACTGGCTGAATGGCAAAGAGTACAAATGTAAGGTAAGCA
ACAAAGCCTTGCCCGCGCCCATCGAAAAGACTATTTCAAAAGCAAAAGGGCAGCCAAGGGAGCCGCAGGTGTATACCCTGCCT
CCTAGCCGGGACGAACTGACAAAAAATCAGGTGAGCCTCACATGCTTAGTAAAAGGTTTTTACCCAAGTGACATAGCTGTTGA
ATGGGAGTCTAACGGACAGCCCGAAAATAACTATAAGACAACACCCCCTGTGCTGGATAGCGACGGCAGTTTCTTTCTGTACT
CAAAGCTCACCGTTGACAAGTCGCGCTGGCAGCAGGGCAACGTGTTTTCTTGCTCCGTGATGCATGAGGCTTTGCACAACCAT
TATACACAGAAAAGTCTTAGCCTCTCCCCTGGAAGCGGCCTGAACGATATCTTCGAGGCTCAGAAGATTGAATGGCACGAGTA
A

MDWTWRVFCLLAVAPGAHSSQVQLVESGGGLVQPGGSLRLSCVVSGTGFTFSKSPMSWARQAPGKEREWVSAIFADSSTYYSD
SVRGRFTISRDNAKNTVYLQMNNVKPEDTAVYYCGHRRLGKTTYDYRGKGTRVTVSSAEAAAKEAAAKEAAAKEAAAKALEAE
AAAKEAAAKEAAAKEAAAKASQMQFVESGGGLVQPGGSLRLSCVVSGTGFTFSKSPMSWARQAPGKEREWVSAIFADSSTYYA
DSVKGRFTISRDNAKNTVYLQMNDVQPEDSAVYYCGHRRLGKRTYDYRGQGTPVTVSSGAAPAAAPAKQEAAAPAPAAKAEAP
AAAPAAKASQVQLVESGGGLVQPGGSLRLSCVVSGTGFTFSKSPMSWARQAPGKEREWVSAIFADSSTYYSDSVRGRFTISRD
NAKNTVYLQMNNVKPEDTAVYYCGHRRLGKTTYDYRGKGTRVTVSSAEAAAKEAAAKEAAAKEAAAKALEAEAAAKEAAAKEA
AAKEAAAKASQGQLVESGGGLVQPGGSLRLSCTASGFTFSSYAMTWHRQAPGKERELVALITNDARTRYGDFVKGRFTISRDN
AKNTIYLQMNTLAPEDTALYYCGVSLLGRNYGQHWGKGTRVTVSSGAAPAAAPAKQEAAAPAPAAKAEAPAAAPAAKASQMQF
VESGGGLVQPGGSLRLSCVVSGTGFTFSKSPMSWARQAPGKEREWVSAIFADSSTYYADSVKGRFTISRDNAKNTVYLQMNDV
QPEDSAVYYCGHRRLGKRTYDYRGQGTPVTVSSAEAAAKEAAAKEAAAKEAAAKALEAEAAAKEAAAKEAAAKEAAAKASQVQ
LVESGGGLVQPGGSLRLSCVVSGTGFTFSKSPMSWARQAPGKEREWVSAIFADSSTYYSDSVRGRFTISRDNAKNTVYLQMNN
VKPEDTAVYYCGHRRLGKTTYDYRGKGTRVTVSSDKTHTCPPCPAPELLGGPSVFLFPPKPKDTLMISRTPEVTCVVVDVSHE
DPEVKFNWYVDGVEVHNAKTKPREEQYNSTYRVVSVLTVLHQDWLNGKEYKCKVSNKALPAPIEKTISKAKGQPREPQVYTLP
PSRDELTKNQVSLTCLVKGFYPSDIAVEWESNGQPENNYKTTPPVLDSDGSFFLYSKLTVDKSRWQQGNVFSCSVMHEALHNH
YTQKSLSLSPGSGLNDIFEAQKIEWHE*

6Nb39-hcAb-Avi (Secretion signal – Nb39 - LEA – Nb41 – PAPA - Nb39 – LEA – Nb40 – PAPA – Nb41 – LEA – Nb39 – hinge – hIgG1-Fc – Avi)

ATGGACTGGACATGGCGCGTCTTTTGTCTGTTGGCAGTTGCACCTGGAGCTCACTCAAGCCAGCGGCAGCTGGTTGAGTCTGG
CGGCGGACTAGTACATACTGGCGGGTCCTTGAAATTGTCCTGCGTGCCTAACGGATCTATATTCAACTTCAATCCCATGGGAT
GGTACAGGCAAGTATCAGGACAGCAGCGCGAGCTGGTCGCCACCCTGACGCGAGACGGCGTTGAGAACTATGCTAGTAGCGTA
AAAGGTAGATTCACTATATCCAGGGATAGTGCGAAGAACACATTATACCTGCAAATGACCGATGTGAAACCCGGTGACGCGGC
TGTTTATATTTGTCATGCCAACTATAGAATCGGGAGGAATGACCTACCAGTGTGGGGGAAGGGCACCCCCGTTACAGTGTCAA
GCGCAGAAGCTGCCGCAAAAGAAGCCGCTGCGAAAGAGGCTGCTGCAAAGGAGGCCGCCGCCAAGGCCCTCGAAGCCGAAGCG
GCCGCCAAAGAGGCTGCAGCTAAGGAAGCAGCCGCCAAAGAGGCAGCTGCAAAAGCCTCTCAGCGCCAGCTCGTAGAAAGCGG
GGGGGGGTCAATGCAACCCGGAGAAAGCCTCACCTTAAGCTGCGAGGCTAGCGATAATATCCTCCAATTCGGCAATATGGGGT
GGTACCGACAGAGTCCGGGGACGCAGCGCGAGCTCGTGGCTCGTATACACAAGCGGGGAGATTCTGACTACGGAGACTTCGCT
AAAGGAAGATTCACAATCTCAAGAGATACTGTCAAAAATAAAGTCTATCTCCAGATGACAGACCTTAAGCCGGAGGACTCAGC
GAATTACATCTGCAATGGACAGTATGTCATCGGCCGGAATCGTCTGGACGTTTGGGGGCAGGGGACACCTGTAACTGTCTCCA
GCGGTGCTGCACCAGCGGCAGCACCTGCCAAGCAGGAGGCCGCAGCTCCTGCACCGGCCGCAAAGGCTGAAGCCCCGGCGGCC
GCTCCCGCCGCCAAGGCCTCGCAGAGGCAACTCGTGGAATCGGGAGGGGGACTTGTCCACACCGGCGGTTCCCTTAAATTATC
ATGCGTGCCCAACGGCTCTATTTTTAACTTCAACCCTATGGGCTGGTACCGCCAGGTCTCCGGACAGCAGAGGGAGCTGGTGG
CGACCCTCACCCGTGATGGTGTAGAGAACTATGCCTCTTCCGTGAAAGGTCGGTTCACCATCTCACGAGACTCCGCCAAAAAC
ACTCTCTACCTGCAGATGACGGACGTAAAGCCTGGCGATGCAGCCGTTTACATATGCCACGCAAATTATCGGATCGGGCGAAA
CGACTTGCCCGTGTGGGGCAAAGGCACCCCGGTCACTGTCAGCTCAGCCGAGGCCGCCGCAAAGGAGGCGGCAGCCAAGGAAG
CAGCTGCCAAGGAAGCCGCCGCAAAGGCCCTAGAGGCTGAGGCCGCGGCAAAGGAAGCAGCTGCTAAGGAAGCTGCGGCTAAG
GAGGCAGCTGCTAAGGCTTCCCAGCGCCAGCTTGTGGAGTCTGGCGGAGGACTTGTCCAGCCAGGAGGTTCATTACGGCTGTC
GTGCGTGCCGAACGGAAGTATCTTTAATTTTAACATAATGGGCTGGTATAGACAGAACGCTGGCAACCAGCGCGAGCTTGTTG
CCACCATGACTCGGGACGGGTCTGCGTCTTACAGCGATTCTGTGAAAGGCCGCTTCACCATATCCCGCGACGTGGACAAGAAC
ACTATCTACCTGCAACTGGACTCCGTAAAACCAGAAGATACAGCTGTGTACATTTGTCATGCGAACTATAGAATCGGAAGGAA
TGACCTCCCTGTATGGGGGAGGGGTACACGAGTTACCGTGTCCTCTGGGGCCGCGCCAGCCGCTGCTCCTGCCAAGCAGGAGG
CTGCAGCTCCTGCCCCCGCTGCAAAGGCTGAAGCACCGGCCGCTGCGCCCGCTGCGAAGGCCTCTCAGCGGCAGCTGGTTGAA
TCTGGAGGAGGAAGTATGCAGCCGGGGGAAAGCCTCACACTTTCATGTGAGGCAAGCGACAATATCCTGCAGTTTGGCAATAT
GGGCTGGTATCGGCAATCGCCTGGAACCCAGAGAGAACTGGTTGCCAGGATACACAAGAGGGGGGATTCCGATTATGGGGACT
TTGCGAAGGGGAGATTCACTATTTCTCGGGATACTGTGAAGAATAAAGTGTATCTTCAAATGACTGACCTTAAACCAGAAGAT
AGTGCGAACTATATCTGTAACGGCCAATATGTTATCGGGAGAAATAGACTCGATGTCTGGGGGCAGGGTACCCCAGTCACCGT
CAGTTCCGCCGAGGCCGCCGCTAAGGAGGCCGCCGCCAAAGAAGCGGCCGCTAAAGAAGCAGCCGCCAAAGCGCTGGAGGCTG
AGGCCGCCGCTAAGGAAGCTGCCGCCAAGGAAGCGGCCGCAAAAGAGGCTGCTGCCAAGGCCTCCCAGCGTCAATTGGTGGAA
TCAGGAGGCGGGCTCGTCCACACTGGCGGCTCTCTTAAATTGTCATGCGTGCCTAACGGTTCCATTTTTAACTTTAATCCGAT
GGGCTGGTATCGGCAGGTTTCTGGCCAGCAGAGGGAACTTGTCGCAACACTGACAAGAGATGGGGTTGAAAATTATGCCAGCA
GCGTTAAGGGGAGATTCACCATATCTCGCGACAGCGCAAAGAATACACTGTATCTACAGATGACCGATGTTAAGCCCGGCGAC
GCTGCTGTTTACATCTGCCACGCCAATTATCGTATTGGTCGCAACGATCTGCCTGTGTGGGGAAAAGGAACGCCAGTTACCGT
GTCGAGCGATAAGACTCATACTTGTCCTCCATGTCCGGCCCCAGAATTACTCGGAGGACCAAGTGTCTTTCTGTTCCCACCCA
AACCCAAGGACACCCTGATGATATCGCGAACCCCGGAGGTGACATGTGTGGTGGTTGACGTTAGCCACGAGGACCCAGAGGTG
AAGTTTAACTGGTACGTTGATGGCGTTGAAGTACATAATGCTAAAACCAAGCCTAGAGAGGAGCAGTATAACTCAACTTACCG
AGTGGTCTCTGTGCTGACCGTGCTGCACCAGGATTGGCTTAACGGTAAGGAGTACAAGTGTAAAGTGTCTAATAAAGCGCTCC
CTGCGCCAATTGAGAAGACTATATCTAAGGCAAAAGGGCAGCCCCGAGAACCTCAAGTGTACACATTGCCCCCGTCACGTGAC
GAGCTGACCAAGAATCAGGTGTCCCTCACCTGTCTCGTGAAGGGCTTTTATCCTTCTGACATCGCAGTCGAGTGGGAATCCAA
CGGGCAGCCCGAAAACAACTACAAAACCACACCACCCGTTCTCGACTCAGACGGGTCTTTCTTCCTTTATTCAAAGTTGACCG
TTGATAAAAGTCGCTGGCAGCAGGGCAATGTGTTCTCCTGCAGCGTGATGCACGAAGCCCTGCACAACCATTATACCCAGAAG
AGCCTGAGTCTCAGTCCGGGATCTGGCCTGAATGATATATTTGAAGCACAGAAAATCGAGTGGCACGAGTAA

MDWTWRVFCLLAVAPGAHSSQRQLVESGGGLVHTGGSLKLSCVPNGSIFNFNPMGWYRQVSGQQRELVATLTRDGVENYASSV
KGRFTISRDSAKNTLYLQMTDVKPGDAAVYICHANYRIGRNDLPVWGKGTPVTVSSAEAAAKEAAAKEAAAKEAAAKALEAEA
AAKEAAAKEAAAKEAAAKASQRQLVESGGGSMQPGESLTLSCEASDNILQFGNMGWYRQSPGTQRELVARIHKRGDSDYGDFA
KGRFTISRDTVKNKVYLQMTDLKPEDSANYICNGQYVIGRNRLDVWGQGTPVTVSSGAAPAAAPAKQEAAAPAPAAKAEAPAA
APAAKASQRQLVESGGGLVHTGGSLKLSCVPNGSIFNFNPMGWYRQVSGQQRELVATLTRDGVENYASSVKGRFTISRDSAKN
TLYLQMTDVKPGDAAVYICHANYRIGRNDLPVWGKGTPVTVSSAEAAAKEAAAKEAAAKEAAAKALEAEAAAKEAAAKEAAAK
EAAAKASQRQLVESGGGLVQPGGSLRLSCVPNGSIFNFNIMGWYRQNAGNQRELVATMTRDGSASYSDSVKGRFTISRDVDKN
TIYLQLDSVKPEDTAVYICHANYRIGRNDLPVWGRGTRVTVSSGAAPAAAPAKQEAAAPAPAAKAEAPAAAPAAKASQRQLVE
SGGGSMQPGESLTLSCEASDNILQFGNMGWYRQSPGTQRELVARIHKRGDSDYGDFAKGRFTISRDTVKNKVYLQMTDLKPED
SANYICNGQYVIGRNRLDVWGQGTPVTVSSAEAAAKEAAAKEAAAKEAAAKALEAEAAAKEAAAKEAAAKEAAAKASQRQLVE
SGGGLVHTGGSLKLSCVPNGSIFNFNPMGWYRQVSGQQRELVATLTRDGVENYASSVKGRFTISRDSAKNTLYLQMTDVKPGD
AAVYICHANYRIGRNDLPVWGKGTPVTVSSDKTHTCPPCPAPELLGGPSVFLFPPKPKDTLMISRTPEVTCVVVDVSHEDPEV
KFNWYVDGVEVHNAKTKPREEQYNSTYRVVSVLTVLHQDWLNGKEYKCKVSNKALPAPIEKTISKAKGQPREPQVYTLPPSRD
ELTKNQVSLTCLVKGFYPSDIAVEWESNGQPENNYKTTPPVLDSDGSFFLYSKLTVDKSRWQQGNVFSCSVMHEALHNHYTQK
SLSLSPGSGLNDIFEAQKIEWHE*

Supplementary Figure 14 | DNA sequences of cloned nanobodies constructs and the resulting protein sequence. Construct names are highlighted in bold. Color codes and shades are indicated on top.

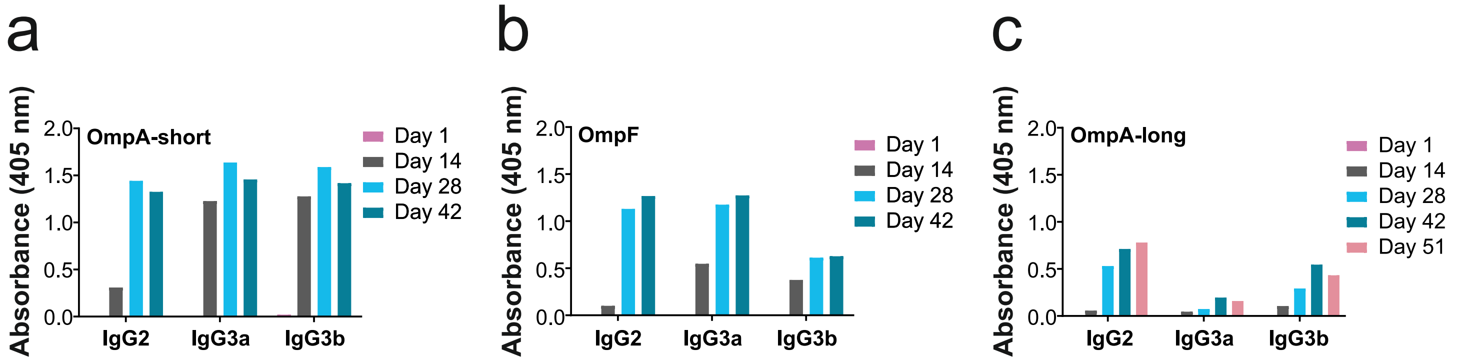

Supplementary Figure 15 | Serum ELISA of heavy chain only antibody response against respective target proteins. Blood serum samples of immunized Alpacas were analyzed for the binding signal against OmpA-short (**a**), OmpF (**b**) and OmpA-long (**c**). Blood samples used to generate ELISAs shown (**a**) and (**b**) stem from the same Alpaca, which was co-immunized with OmpA-short and OmpF, while a separate Alpaca was immunized with OmpA-long (**c**).
