# Supplementary material for: Rapid detection and capture of clinical Escherichia coli strains mediated by OmpA-targeting nanobodies
Source: Commun Biol. 2025 Jul 14;8:1047. doi: 10.1038/s42003-025-08345-9 (PMC12259851; doi:10.1038/s42003-025-08345-9)
Supplement: Supplementary file 3 — Description of Additional Supplementary Files [file 42003_2025_8345_MOESM3_ESM.pdf]

Description of Additional Supplementary Files

File name: Supplementary Data
Description: The source data behind the graphs in the figures
